# Structural basis for oxidation-coupled isomerization by ApnO

**DOI:** 10.1016/j.jbc.2026.113628

**Published:** 2026-10-09

**Authors:** Seiya Watanabe, Yuzuki Aono, Shin-ichi Terawaki, Himika Sato, Taiyo Yokoi

**Affiliations:** 1 Department of Bioscience, Graduate School of Agriculture, Ehime University, Matsuyama, Ehime, Japan; 2 Faculty of Agriculture, Ehime University, Matsuyama, Ehime, Japan; 3 Center for Marine Environmental Studies (CMES), Ehime University, Matsuyama, Ehime, Japan; 4 Division of Structure Analysis of Protein Complex, Proteo-Science Center (PROS), Ehime University, Matsuyama, Ehime, Japan

**Keywords:** anomalous dispersion analysis, binuclear metal center, D-apionate oxidoisomerase, gene cluster, ketol-acid reductoisomerase

## Abstract

D-Apionate oxidoisomerase (ApnO), involved in D-apiose metabolism from bacteria, is one of the few examples of the enzymes that catalyze two different reactions within a single active site, whereas there is no functional orthologue with significant sequence identity. Based on genome context, we found that ApnO additionally utilizes D-erythronate and D-ribonate as substrates, which made it possible to functionally characterize the enzyme. Crystallographic studies (using anomalous dispersion analysis) revealed that the overall folding of ApnO was similar to that of ketol-acid reductoisomerase (KARI), with two magnesium ions at sites II and I, and that the purified (inactive) enzyme contained one zinc ion at site II (and NAD^+^). Further crystallization in the presence of manganese ion led to the binding of manganese ions at both sites II and I. Among them, the coordination geometry at site II was uniquely tetrahedral, differed from the typical octahedral geometry observed at site I. Several crystal structures in complex with malonate and/or its derivative inhibitor, together with substrate docking simulations, showed a mechanism of oxidation-coupled isomerization similar to that of KARI *via* a cyclopropane transition state, whereas only four active site residues around the metal ion binding site I were conserved between ApnO and KARI, suggesting that they convergently acquired the second metal ion binding site (site II) in evolutional stage.

Seven EC classes of enzymes, developed by the International Commission on Enzymes (EC) and adopted by the International Union of Biochemistry and Molecular Biology (IUBMB), are widely used for reaction-based classification. On the other hand, a few enzymes cannot be unambiguously assigned to a single class, and are classified into three groups (1). The first group comprises well-known multifunctional enzymes, such as fatty acid synthase and tryptophan synthase. They consist of two or more subunits (or domains), in which different reactions occur within each active site. Ribulose-1,5-bisphosphate carboxylase/oxygenase is a typical example of the second group that performs distinct reactions within a single active site, independently of each other. The third group, which is the focus of this study, comprises enzymes that catalyze two reactions sequentially within the same active site. They include four unique isomerases that couple isomerization activity with an EC 1 reaction class activity (Fig. S1): aurachin C monooxygenase/isomerase (2); 1-deoxy-D-xylulose 5P reductoisomerase (3); ketol-acid reductoisomerase (EC 1.1.1.86; KARI) (4); D-apionate oxidoisomerase (EC 1.1.1.421; ApnO) (5). Among them, only ApnO is physiologically involved in catabolism (but not anabolism) and has not been subjected to detailed structure-function analysis.

An origin of D-apionate in nature is D-apiose, a widely distributed *C*-branched pentofuranose in higher plants such as parsley and celery (6), which is synthesized from UDP-D-glucuronate as UDP-D-apiose, the building block of cell wall biosynthesis (7). In 2018, Gerlt and coworkers (5) first reported four classified pathways of D-apiose/D-apionate metabolism in bacteria, among which oxidative routes 1∼3 contain a reaction step by ApnO, which converts D-apionate to 3-oxo-D-isoapionate (Fig. 1*A*). Although these metabolic genes (enzymes) are frequency clustered on the bacterial genome (Fig. 1, *D*, *E* and *G*) and mostly belong to an uncharacterized protein family, there has been no further biochemical or structural analysis since 2018, probably because they didn’t describe a practical method for preparing the commercially unavailable D-apiose and D-apionate in sufficient quantities for functional characterization.

**Figure 1:**
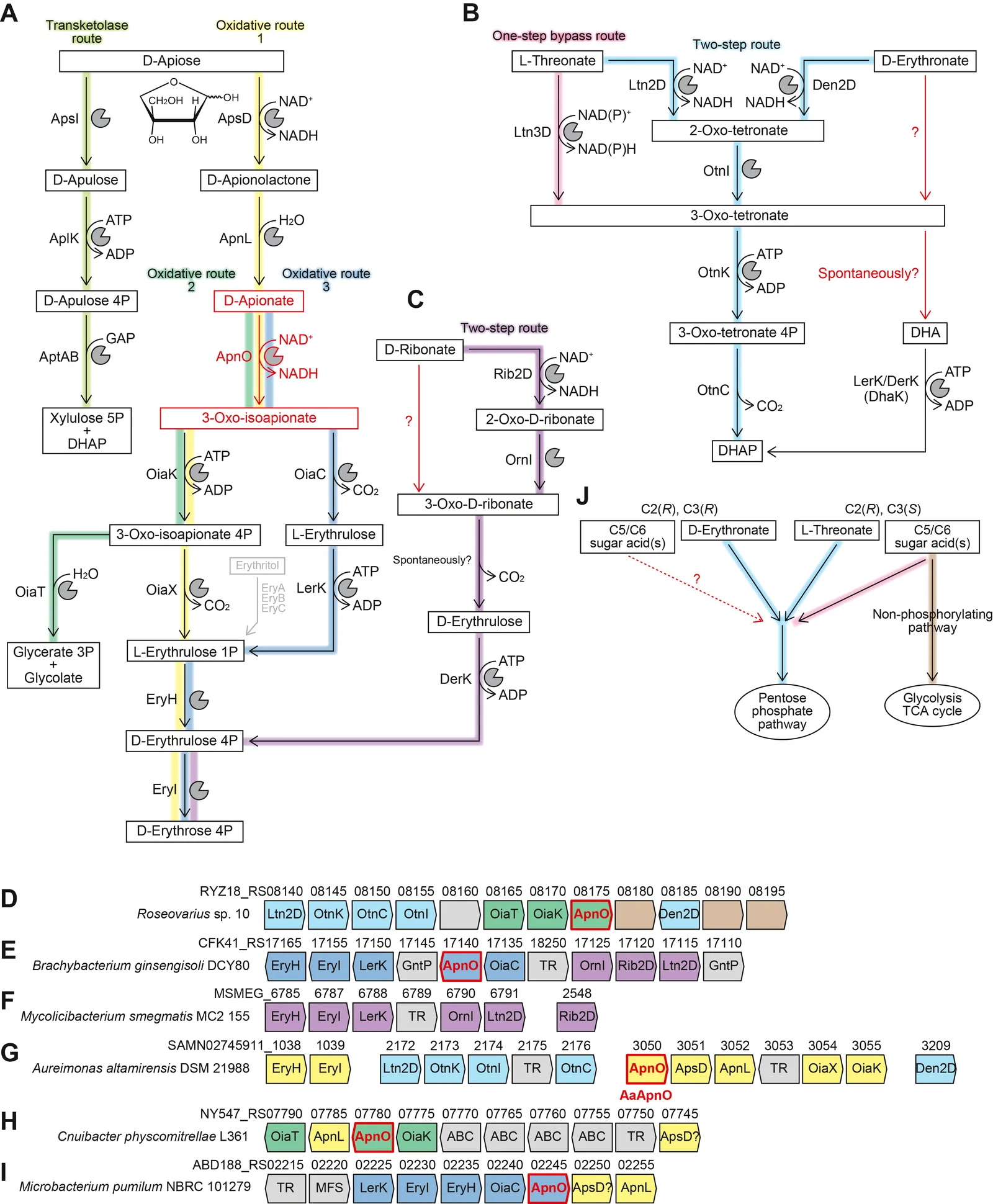
**Microbial metabolism of sugar acids.***A*, metabolic pathways of D-apiose and D-apionate. These are classified into one transketolase route (*lime*) and three oxidative routes (*yellow*, *green*, *and blue*), among which the latter commonly contain D-apionate intermediate as the substrate for ApnO. The inset indicates the chemical structure of D-apiose, a branched monosaccharide. Metabolic pathways of C4 sugar acids (*B*) and D-ribonate (*C*). In each two-step route (*cyan and purple*), sugar acids are converted to 3-oxo acids *via* 2-oxo acids by sequential reactions of a dehydrogenase and an isomerase. Our recently identified Ltn3D corresponds to a bypass route of L-threonate (*pink*). We elucidated whether there is a bypass route for D-erythronate and D-ribonate by ApnO (*red*) or not. *D*–*I*, schematic gene clusters related to C4/C5/C6 sugar acids, D-ribonate, and/or D-apiose/D-apionate metabolism from bacteria. Gene components are indicated in the same color and correspond to Figure 1, *A–C*. Genes in *gray* are putative transcriptional regulators and/or transporters. AaApnO gene in Figure 1*G* was functionally characterized in this study. *J*, metabolic crosstalk between C4/C5/C6 sugar acids. Metabolic pathways are indicated in the same colors as Figure 1, *A–C*. ApnO, D-Apionate oxidoisomerase; Ltn3D, L-threonate 3-dehydrogenase.

Our laboratory has been working to identify and functionally and structurally characterize novel metabolic enzyme(s) related to the so-called non-phosphorylating pathway of C5/C6 sugar and/or sugar acid metabolism, which is (partially) homologous to the unique glycolytic pathway from archaea (8). The utilization of C4 sugar acids (tetronates), including D- and L-threonate and D-erythronate was also reported by Gerlt and coworkers in 2016 at the molecular level (9). In the two-step route related to this study (cyan in Fig. 1*B*), L-threonate and D-erythronate are converted to 2-oxo-tetronate by different dehydrogenases, Ltn2D and Den2D, respectively. The 2-oxo-tetronate intermediate is further metabolized to dihydroxyacetone phosphate (DHAP) *via* 3-oxo-tetronate and 3-oxo-tetronate 4P by an isomerase (OtnI), a kinase (OtnK), and a decarboxylase (OtnC).

We recently found that, in unusual gene clusters related to C4/C5/C6 sugar acid metabolism from bacteria, a hypothetical short-chain dehydrogenase/reductase-like gene (protein) encodes a novel NAD(P)^+^-dependent L-threonate 3-dehydrogenase (Ltn3D) (10). This reaction corresponds to the one-step bypass route for the conversion of L-threonate to 3-oxo-L-threonate (3-oxo-tetronate) by Ltn2D and OtnI (pink in Fig. 1*B*). Furthermore, in contrast to Ltn2D, Ltn3D utilizes not only L-threonate but also other C5/C6 sugar acids with C2(*R*) and C3(*S*) hydroxyl groups (in particular, D-gluconate) (Fig. 1*J*), which may provide an evolutionary advantage by enabling co-metabolism of C4/C5/C6 sugar acids within a single gene cluster and/or direct entry into the pentose-phosphate pathway, but not glycolysis and/or the TCA cycle for non-phosphorylating pathway. On the other hand, this finding raised the question of why only L-threonate has a bypass route *via* Ltn3D and whether other C5/C6 sugar acid(s) with C2(*R*) and C3(*R*) hydroxyl groups is also co-metabolized.

We herein found that, based on genome context analysis, ApnO utilizes not only D-apionate but also other (easily available) sugar acids; its reaction with D-erythronate corresponds to a bypass route mediated by Den2D and OtnI (red in Fig. 1*B*). An X-ray crystallographic study (using anomalous dispersion analysis) revealed that ApnO had a similar structural framework to KARI, one of the unique double-duty isomerases, whereas the binuclear metal center was significantly different between them. Furthermore, crystal structures in complex with a coenzyme and an inhibitor provided the important insight into the oxidation-coupled isomerization mechanism, which is fundamentally similar to KARI, whereas it appears to have evolved convergently.

## Results

### Genome context analysis of D-apiose and D-apionate pathways

Although it had been believed that there is no metabolic crosstalk between D-apiose/D-apionate and other carboxylate pathways, the (ApnO-containing) metabolic genes related to oxidative routes 2 and 3 of D-apiose/D-apionate pathway are located near gene clusters for C4 and C5 (D-ribonate) sugar acid metabolism, respectively, on the genomes of several bacteria (Fig. 1, *D* and *E*). In the latter case, D-ribonate is converted to D-erythrulose 4P by a dehydrogenase (Rib2D), an isomerase (OrnI), and an unidentified decarboxylase (or spontaneously), and a kinase (DerK) (11) (Fig. 1, *C* and *F*). It is noteworthy that the same metabolic fate is also found in oxidative route 3 of the D-apiose/D-apionate pathway. Therefore, based on this genome context analysis, we assumed that ApnO may utilize additional sugar acid(s) except D-apionate as a substrate. To test this hypothesis, the (putative) ApnO gene from *Aurantimonas altamirensis* (AaApnO), a soil-dwelling bacterium (12), was selected as a target protein (Uniprot ID: A0ABY1INU2; Locus: SAMN02745911_3050), and showed significant sequence identities to four ApnO enzymes, identified by Gerlt and coworkers (5) (see Table 1). This bacterium separately possesses gene clusters related to oxidative route 1 of D-apiose metabolism and two-step route of C4 sugar acid metabolism (Fig. 1*G*).

**Table 1:** Kinetic parameters of AaApnO

Enzyme	Homology (%)	Substrates	Coenzyme	Metal ion	K_m_ (mM)	k_cat_ (min^−1^)	k_cat_/K_m_ (min^−1^ mM^−1^)	Reference
AaApnO (A0ABY1INU2)	(100)	D-Erythronate	NAD^+^	Mn^2+^	0.882 ± 0.118^a^	108 ± 9	123 ± 6	This study
		L-Lyxonate			0.655 ± 0.059^a^	127 ± 7	195 ± 7	
		D-Ribonate			0.743 ± 0.040^a^	203 ± 5	274 ± 7	
		L-Mannonate			1.83 ± 0.85^b^	12.9 ± 2.0	7.64 ± 2.44	
		D-Gulonate			4.87 ± 0.24^b^	163 ± 4	33.5 ± 0.9	
		L-Rhamnonate			1.68 ± 0.16^a^	452 ± 30	269 ± 8	
		Hydroxypyrubate	NADH		0.490 ± 0.033^a^	14.1 ± 0.4	28.7 ± 1.0	
		Oxomalonate			0.708 ± 0.025^a^	70.4 ± 2.9	99.4 ± 2.0	
B9JK75	80	D-Apionate	NAD^+^		0.15 ± 0.01	78.0 ± 1.2	520	(5)
F8GV06	74				0.27 ± 0.03	29.4 ± 0.6	109	
Q6D8V3	39				0.13 ± 0.01	28.2 ± 0.6	217	
C0CMQ7	46				0.25 ± 0.03	19.2 ± 0.6	76.8	

Values are the means ± SD, *n* = 3.

a Eight different substrate concentrations between 0.1 and 1 mM were used.

b Eight different substrate concentrations between 1 and 10 mM were used. ApnO, D-Apionate oxidoisomerase.

**Table 2:** Metal ion specificity of AaApnO

Reaction	Substrate	Metal ion	K_m_ (mM)	k_cat_ (min^−1^)	k_cat_/K_m_ (min^−1^ mM^−1^)
Oxidation	D-Ribonate	Mn^2+^	0.743 ± 0.040^a^	203 ± 5	274 ± 7
		Mg^2+^	4.03 ± 0.50^a^	61.3 ± 5.6	15.3 ± 0.5
		Ni^2+^	N.D.^b^	N.D.	N.D.
		Co^2+^	N.D.	N.D.	N.D.
		Ca^2+^	N.D.	N.D.	N.D.
Reduction	Hydroxypyruvate	Ni^2+^	0.520 ± 0.021^a^	31.4 ± 0.9	60.4 ± 0.8
		Mn^2+^	0.490 ± 0.033^a^	14.1 ± 0.4	28.7 ± 1.0
		Co^2+^	1.91 ± 0.11^a^	45.9 ± 2.2	24.0 ± 0.3
		Mg^2+^	2.82 ± 0.16^a^	2.66 ± 0.12	0.943 ± 0.11
		Ca^2+^	N.D.	N.D.	N.D.

Values are the means ± SD, *n* = 3. ApnO, D-Apionate oxidoisomerase.

a Eight different substrate concentrations between 0.1 and 2 mM were used under 1 mM metal ion.

b Not assessed due to trace activity.

### Functional characterization of AaApnO

Library of C4/C5/C6 sugar acids (excluding D-apionate) were screened as potential substrates for the recombinant AaApnO protein (Fig. S2). Among them, strictly NAD^+^-dependent oxidation activity was observed for several sugar acids with the same C2(*R*) and C3(*R*) hydroxyl group configuration as D-apionate: D-erythronate (C4), D-ribonate (C5), L-lyxonate (C5), L-mannonate (C6), D-gulonate (C6), and L-rhamnonate (C6). D-Allonate (C6) and L-talonate (C6) were not tested (Fig. 2*A*). The optimum pH was 10 using glycine-NaOH buffer, and the activities at pH 8 and 9 using Tris-HCl buffer were 18% and 39% of the maximum activity, respectively; Gerlt and coworkers assayed the enzyme in Tris-HCl buffer at pH 9 (5). Importantly, these compounds are readily available, which made it possible to carry out a detailed functional characterization.

**Figure 2:**
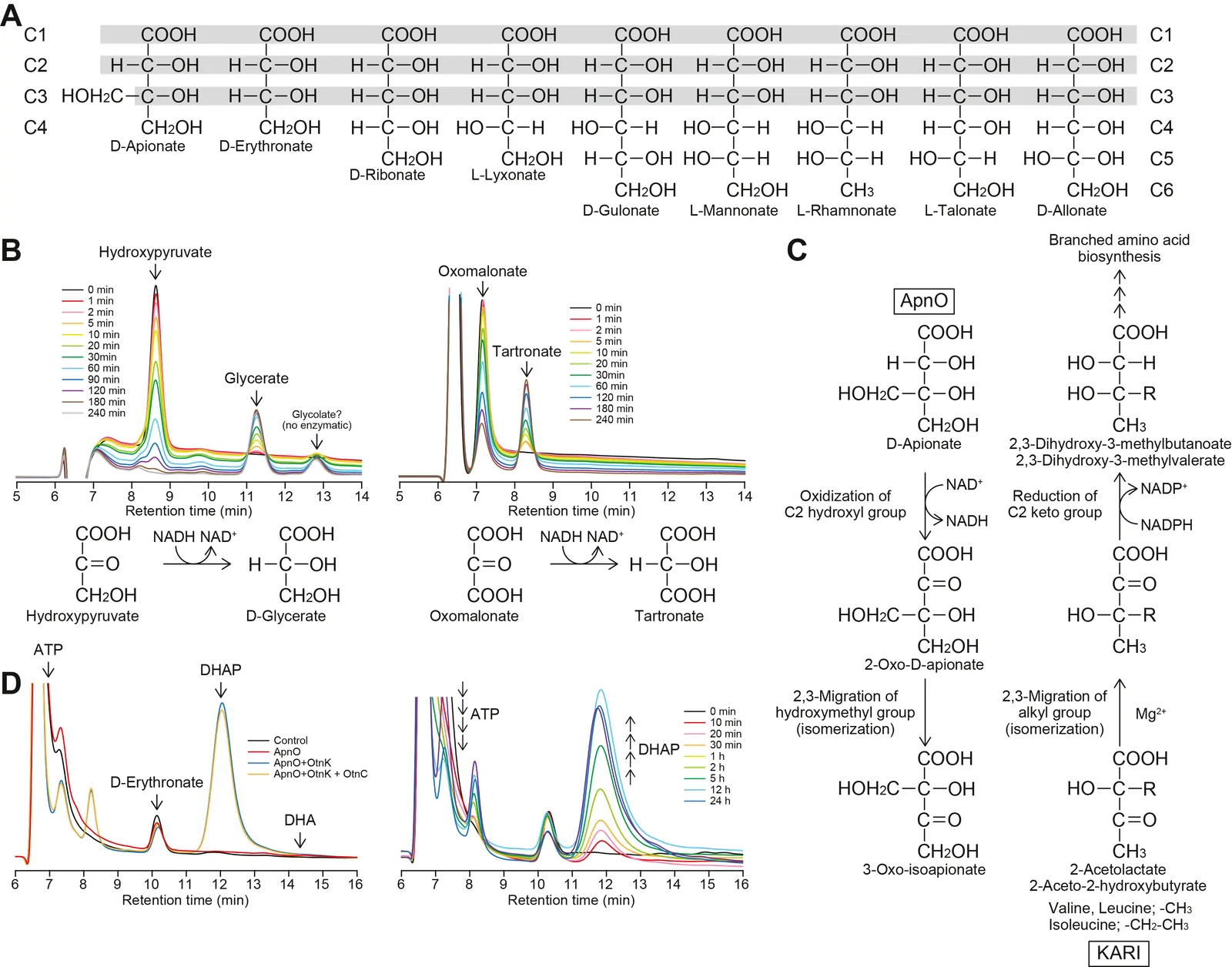
**Biochemical characterization of AaApnO.***A*, configurations of substrates. Structural formula of all tested C4/C5/C6 sugar acids for substrate screening is shown in Fig. S2. The *gray-shaded* configuration is identical to that of D-apionate. D-Allonate and L-talonate were not tested. *B*, HPLC analysis of reaction products formed by NADH-dependent reduction of hydroxypyruvate (*left*) and oxomalonate (*right*). It is known that hydroxypyruvate is no enzymatically degraded to other compounds such as glycolate (50), which may correspond to a peak with 12.8 min of retention time. *C*, schematic reaction of ApnO proposed by Gerlt and coworkers (5) and its comparison with KARI. *D*, HPLC analysis of sequential reactions by ApnO and 3-oxo-tetronate 4P a kinase (and 3-oxo-tetronate 4-phosphate decarboxylase) from D-erythronate in the presence of NAD^+^ and ATP. The elution profile of each component is shown in Fig. S4. ApnO, D-Apionate oxidoisomerase.

Since the purified AaApnO protein was completely inactive, this dehydrogenase activity was initially measured in the presence of Mn^2+^ or Mg^2+^, as described by Gerlt and coworkers (5). Although they did not report a preference between these metal ions, we found that the *k*_cat_/*K*_m_ value in the presence of Mn^2+^ (using D-ribonate as the substrate) was approximately 18-fold higher than that obtained in the presence of Mg^2+^; no activity was observed in the presence of Ni^2+^, Co^2+^, Fe^2+^, Zn^2+^, Fe^2+^, or Ca^2+^ (Table 2). Accordingly, the kinetic parameters for the substrates were determined in the presence of Mn^2+^. The *k*_cat_/*K*_m_ values for the C4/C5/C6 sugar acids, except L-mannonate and D-gulonate, were comparable to those reported for D-apionate with known ApnO enzymes (5) (Table 1).

Although the corresponding 2-oxo acids derived from these active sugar acids were not available, hydroxypyruvate and oxomalonate were active substrates for the NADH-dependent reductive reaction, and their *k*_cat_/*K*_m_ values were similar to those observed for the oxidative reaction (28.7 and 99.4 min^−1^ mM^−1^, respectively; Table 1). Unexpectedly, in this reverse (reductive) direction, significant activity was found in the presence of not only Mn^2+^ and Mg^2+^ but also Ni^2+^ and Co^2+^ (Table 2). Among them, the *k*_cat_/*K*_m_ value in the presence of Mg^2+^ was 25∼64-fold lower than those obtained with the other metal ions. In the HPLC analysis, when AaApnO was incubated with hydroxypyruvate or oxomalonate in the presence of NADH (and Mn^2+^), novel peaks with retention times of 11.3 min and 8.2 min appeared in a time-dependent manner and were identified as glycerate and tartronate, respectively (Fig. 2*B*). These results indicated that reaction by AaApnO includes a reversible oxidoreduction reaction at the C2 hydroxyl group (at least).

### Elucidation of oxidative reaction

Gerlt and coworkers (5) proposed that the reaction mechanism of ApnO involves oxidation of the C2 hydroxyl group of D-apionate, following by migration of the hydroxyl methyl group, similar to KARI (Fig. 2*C*). When this scheme was applied to the active substrates identified in this study, there were three possibilities (1): a 3-oxo acid is produced through the same mechanism (2); a 2-oxo acid is produced, because no proton migration occurs (3); a 3-oxo acid is produced by directly oxidizing the C3 hydroxyl group, similar to Ltn3D (Fig. S3).

Coupled-enzyme assays were performed using both HPLC and spectrophotometric analyses. In the HPLC analysis, when D-erythronate was incubated with OtnK in the presence of NAD^+^ and ATP, a novel peak appeared in a time-dependent manner, concomitant with decreases in ATP (Fig. 2*D*). Its retention time (∼12 min) was close to that of DHAP (but not that of DHA) (Fig. S4); the further addition of OtnC had no effect on the elution profile (Fig. 2*D*). In the spectrophotometric assay (10), the oxidation of D-erythronate was monitored in the presence of AaApnO, OtnK, OtnC, ATP, and NAD^+^ (Fig. S5*A*). The addition of NADH-dependent glycerol 3P dehydrogenase (G3PDH) significantly decreased the absorbance, which was caused by oxidation of NADH by G3PDH (*i.e.*, the reduction of DHAP to produce glycerol 3P). Collectively, these results suggest that DHAP was produced from D-erythronate through sequential reactions catalyzed by ApnO, OtnK, and OtnC in the absence of an isomerase (OtnI). On the other hand, this may still not be sufficient to provide evidence that the 3-oxo-D-erythronate was indeed produced only through the first possibility, although ApnO showed reductive activity toward the 2-oxo acid(s) (Fig. 2*B*), unlike Ltn3D (10). In this regard, the third possibility was completely excluded by crystal structure(s) and molecular docking model, described below.

When dihydroxyacetone kinase (DhaK) was used instead of OtnK, a similar decrease in absorbance was also observed (Fig. S5*B*), suggesting that the reaction product by ApnO, 3-oxo-D-erythronate, is partially spontaneously decarboxylated to DHA, unlike 3-oxo-L-threonate produced by Ltn3D (Fig. 1*B*).

### Overall structure of AaApnO

To identity the reaction mechanism at the atomic level, we carried out X-ray crystallographic analysis of AaApnO. To investigate the requirement for metal ions (Table 2), the recombinant (inactive) AaApnO was initially crystallized under the condition of Index G7 (Hampton) in the absence of metal ions. The structure was determined by the molecular replacement method using the structure predicted by *AlphaFold2* (13) as the search mode, and subsequently refined to a resolution of 1.44 Å in the *P*2_1_2_1_2_1_ space group with two molecules in the asymmetric unit (PDB ID 43IC), yielding sufficient stereochemical quality. Data processing and structure refinement statistics are listed in Table 3.

**Table 3:** Data collection and crystallographic refinement statistics of AaApnO

PDB code	43IC	26JC	26TF	44CF	43IA	26TG	26TH	26MW
Crystallization	Index G7	Index G7	Index H3	Index H3	Index H3	Index H3	Index F2	Index H3
Ligand(s)								
NAD^+^	○	○	○	○	○	○	○	○
Zn(II)	○	○						
Zn(II)/Mn(II)			○	○	○	○	○	○
Mn(I)			○	○		○	○	○
Malonate			○	○	○	○ (covalent bonding)		
CPD							○	
Data collection								
Space group	*P*2_1_2_1_2_1_	*P*2_1_2_1_2_1_	*P*2_1_2_1_2_1_	*P*2_1_2_1_2_1_	*P*2_1_2_1_2_1_	*P*2_1_2_1_2_1_	*P*2_1_2_1_2_1_	*P*2_1_2_1_2_1_
*a,b,c* (Å)	70.89, 75.37, 104.22	71.19, 75.64, 104.40	70.89, 74.66, 103.96	71.49, 75.63, 104.52	71.40, 75.82, 104.62	71.71, 75.79, 104.57	71.10, 74.97, 103.93	71.32, 75.61, 104.64
α,β,γ (°)	90.00, 90.00, 90.00	90.00, 90.00, 90.00	90.00, 90.00, 90.00	90.00, 90.00, 90.00	90.00, 90.00, 90.00	90.00, 90.00, 90.00	90.00, 90.00, 90.00	90.00, 90.00, 90.00
Wavelength (Å)	1.00	1.28	1.28	1.89	1.00	1.00	1.00	1.00
Resolution range (Å)	46.27–1.44 (1.49–1.44)	42.96–1.61 (1.67–1.61)	42.66–1.39 (1.44–1.39)	42.99–2.05 (2.12–2.05)	46.55–1.26 (1.31–1.26)	46.63–1.29 (1.34–1.29)	42.71–1.55 (1.61–1.55)	43.02–1.87 (1.94–1.87)
*R*_merge_	0.258 (3.191)	0.731 (2.634)	0.398 (3.933)	0.137 (0.393)	0.196 (3.719)	0.118 (1.636)	0.590 (4.294)	0.194 (0.638)
*R*_meas_	0.263 (3.249)	0.751 (2.693)	0.422 (4.154)	0.140 (0.404)	0.200 (3.793)	0.120 (1.667)	0.601 (4.374)	0.199 (0.651)
*R*_pim_	0.050 (0.608)	0.163 (0.553)	0.130 (1.329)	0.029 (0.091)	0.039 (0.738)	0.023 (0.320)	0.116 (0.830)	0.041 (0.129)
*CC*_1/2_	0.998 (0.646)	0.804 (0.691)	0.867 (0.465)	0.996 (0.987)	0.999 (0.591)	0.999 (0.833)	0.988 (0.807)	0.992 (0.950)
*I*/σ *I*	12.3 (1.7)	17.5 (6.2)	9.0 (1.6)	31.4 (15.6)	21.1 (1.4)	17.9 (3.4)	12.9 (2.8)	26.5 (19.2)
Completeness (%)	98.68 (97.27)	99.59 (99.71)	98.07 (94.40)	99.88 (99.58)	99.00 (97.43)	99.93 (99.89)	99.97 (100.00)	99.16 (98.52)
Redundancy	27.1 (27.8)	23.6 (23.6)	12.0 (9.3)	24.3 (19.4)	25.8 (24.9)	26.5 (26.8)	27.0 (27.8)	25.3 (25.6)
Refinement								
*R*/*R*_free_	0.1658/0.1883	0.1662/0.1892	0.1746/0.1948	0.1667/0.2017	0.1693/0.1836	0.1605/0.1724	0.1619/0.1849	0.1687/0.2049
No. of atoms								
Protein	4143	4160	4129	4104	4151	4112	4087	4127
Ligands	97	90	106	106	104	106	110	92
Water	595	748	722	618	548	877	641	624
*B*-factors (Å^2^)								
Protein	14.38	10.49	15.55	9.40	12.66	15.20	11.77	8.94
Ligands	15.00	11.56	19.47	16.07	13.05	13.50	14.23	20.04
Water	26.25	18.88	25.91	18.25	23.43	26.71	24.24	19.34
r.m.s. deviations								
Bond lengths (Å)	0.007	0.013	0.009	0.002	0.012	0.010	0.011	0.004
Bond angles (°)	0.92	1.32	1.10	0.57	1.25	1.12	1.08	0.67
Ramachandran plot								
Favored (%)	97.98	97.79	97.43	97.06	97.43	97.61	97.79	97.24
Allowed (%)	2.02	2.21	2.57	2.94	2.57	2.39	2.21	2.76
Outliers (%)	0.00	0.00	0.00	0.00	0.00	0.00	0.00	0.00

Values in parentheses are for the highest-resolution shell.

ApnO, D-Apionate oxidoisomerase.

The native molecular mass of AaApnO, estimated by gel filtration, was ∼65 kDa, indicating the homodimeric structure (theoretical value of 29,722 Da for the subunit). The two molecules in the asymmetric unit were essentially identical, being superimposable with a root-mean-square deviation (r.m.sd) of 0.291 Å over Thr2-Thr275 Cα atoms (Fig. 3*A*). Each monomer is composed of two distinct domains: the larger N-terminal domain (residues Thr2-Val174) and the smaller C-terminal domain (residues Thr175-Thr275) (Fig. 3*B*). The N-terminal domain consists of a seven stranded open, twisted mixed β-sheet (β1-β7) with seven flanking α-helices, located above (α1, α2, α6, and α7) and below (α3, α4, and α5) the β-sheet, forming a Rossmann fold. The C-terminal domain consists of five α-helices (α8-α12) and the loops that connect these helices, forming a “knotted structure” required for dimerization.

**Figure 3:**
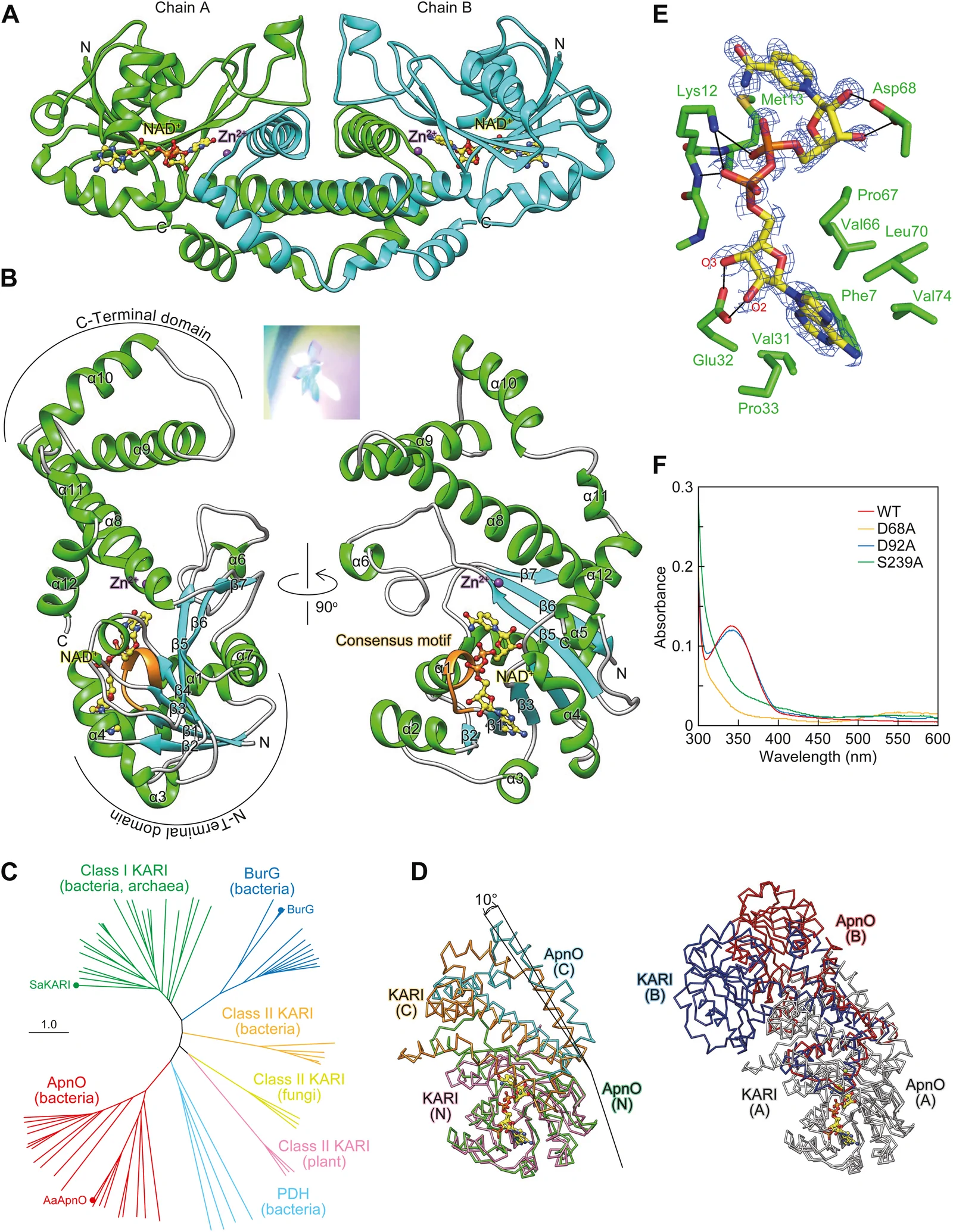
**Overall crystal structure of AaApnO.***A*, ribbon representation of the dimeric assembly. Two subunits *A and B* in the asymmetric unit are colored in *green and cyan*, respectively. Bound NAD^+^ (*yellow*) is represented as a ball-and-stick model. A metal ion, subsequently identified as Zn^2+^, is shown as a *purple* sphere. *B*, ribbon diagram of the subunits. α-Helices and β-strands are colored in *green and cyan*, respectively. The *orange-colored* region corresponds to the characteristic sequence, [Gly/Ala]-X-Gly-X_2_-Gly, of the Rossmann fold motif for dinucleotide binding, corresponding to Figure 7*E*. The inset photo is a crystal of AaApnO. *C*, phylogenetic tree of ApnO and its structural homologs. *Circles* at the end of each branch are used for discussions in the text. An extended tree with a list of sequences and bootstrap values is shown in Fig. S5. *D*, superimposition of the NAD^+^ and Zn^2+^ bound form of dimeric AaApnO with the NADP^+^ and Mg^2+^ bound form of dimeric Class I SaKARI. In the *left panel*, each N-terminal domain (*green and pink*) is fixed, relative to the C-terminal domain (*cyan and orange*). In the *right panel*, each N-terminal domain in subunit A (*gray*) is fixed, relative to subunit *B* (*red and blue*). *E*, electron density of the *F*_o_–*F*_c_ omit map contoured at 1.0 σ, and the model of NAD^+^. The residues responsible for coenzyme recognition are shown as stick models. Hydrogen bonds are shown as *black dashed lines*. *F*, absorption spectra of the purified WT enzyme (*red*), D68A (*orange*), D92A (*blue*), and S239A (*green*) mutants. The concentration of each enzyme solution was 5 mg/ml.

**Figure 4:**
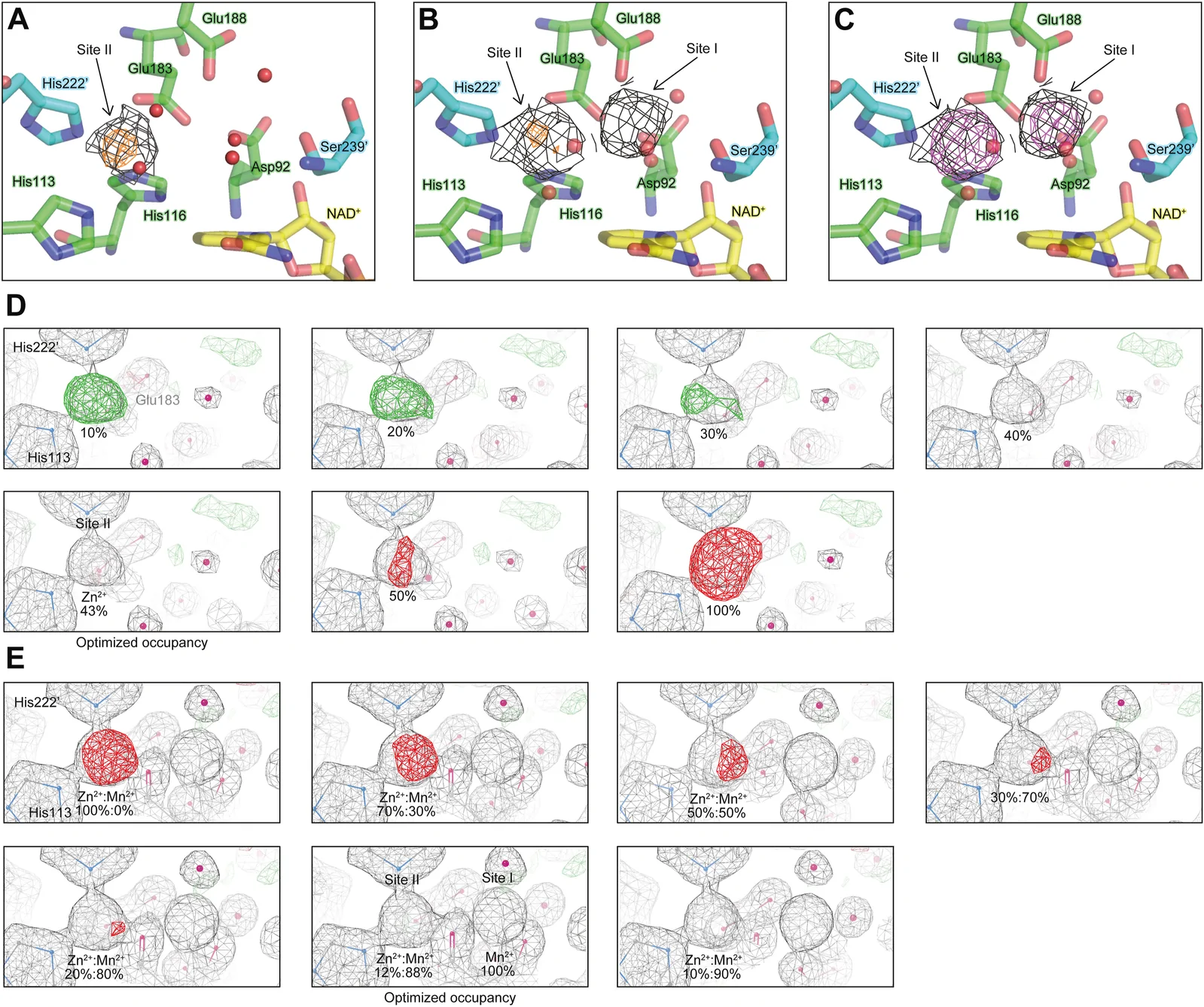
**Identification of the metal center in AaApnO.** Anomalous difference Fourier maps of structures 26JC (*A*) and 26TF (*B and C*) collected at wavelengths of 1.28 Å *(A and B*) and 1.89 Å (*C*) and contoured at 5 σ, are overlaid on the electron density of the *F*_o_‒*F*_c_ omit map, contoured at 1 σ (*black mesh*), corresponding to metal ion(s) at site I and/or II. Anomalous peaks, corresponding to zinc and manganese, are represented by *orange and purple* meshes, respectively. Residues from one and the other subunits are colored in *green and cyan* (primed residues), respectively. 2*F*_o_‒*F*_c_ (*black mesh*) and positive (*green mesh*) or negative (*red mesh*) *F*_o_‒*F*_c_ difference electron density maps of structures 26JC (*D*) and 26TF (*E*) after fitting zinc and/or manganese at site II at the varied occupancies, contoured at 1.5 σ. ApnO, D-Apionate oxidoisomerase.

**Figure 5:**
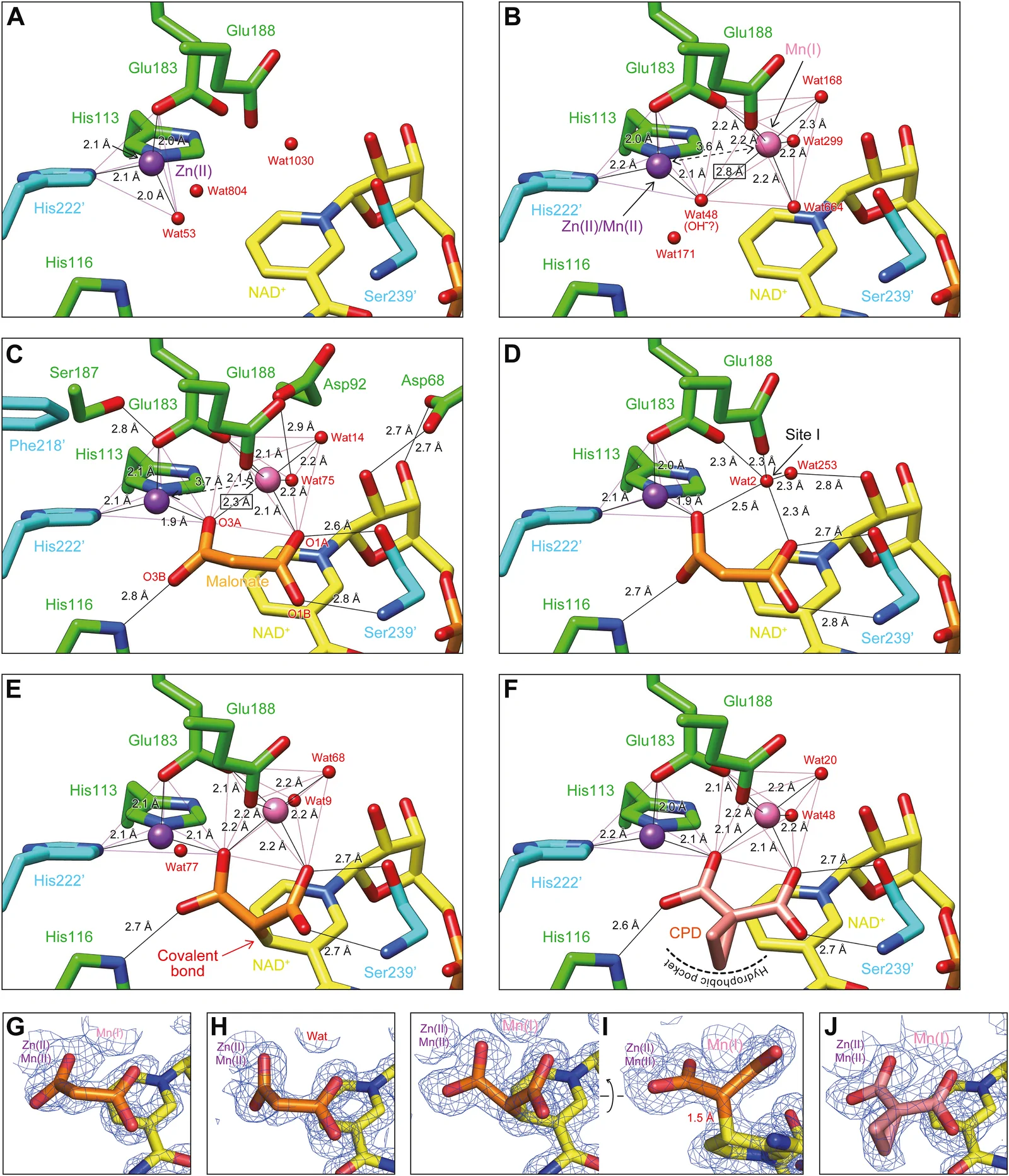
**Metal ion center and ligand binding mode of AaApnO.***A*, Zn(II) bound form (43IC); *B*, Zn(II)/Mn(II) and Mn(I) bound form (26MW); *C*, Zn(II)/Mn(II), Mn(I), and malonate bound form (26TF); *D*, Zn(II)/Mn(II) and malonate bound form (43IA); *E*, Zn(II)/Mn(II), Mn(I), and covalently bound malonate form (26TG); *F*, Zn(II)/Mn(II), Mn(I), and CDP bound form (26TH). *G*–*J*, electron density of the *F*_o_–*F*_c_ omit map, contoured at 1.0 σ, and models of malonate (*G and I*) and CPD (*J*). The figure shows the atomic contacts involved in metal ion and ligand binding and the bond distances (indicated by *black dashed lines*). Schematic tetrahedral and octahedral coordination geometries of the metal ion are shown in *purple* and *pink*, respectively. Residues from one and the other subunits are colored in *green* and *cyan* (primed residues), respectively. ApnO, D-Apionate oxidoisomerase.

A structural similarity search with the DALI server (but not simple Protein-BLAST analysis) revealed that the structure of AaApnO shares a similar fold with ketol-acid reductoisomerase (KARI; 3ULK) (14), cyclopropanol synthase (softwa; 7PCC) (15), prephenate dehydrogenase (5UYY) (16), Δ^1^-pyrroline-5-carboxylate reductase (1YQG) (17), and 6-phosphogluconate dehydrogenase (4GBJ), with r.m.sd values ranging from 3.0 Å to 4.0 Å over approximately 200 Cα atoms. On the other hand, their structural based sequence similarities were uniformly extremely low (∼15%), consistent with the separate clade of the ApnO subfamily in the phylogenetic tree (Figs. 3*C* and S6).

Among these structural homologs, KARI catalyzes an isomerization-coupled reduction reaction within a single active site, in which a binuclear metal center consisting of two magnesium ions is present, as described in “Introduction” (Fig. S1*C*). Importantly, KARI is involved in the biosynthesis of branched-chain amino acids (valine, leucine, and isoleucine). Since this pathway is present only in plants, bacteria, archaea, and fungi (but not mammals), KARI has been extensively studied as a target for antimicrobial and herbicidal agent development, and many crystal structures have been deposited in PDB (Table S1). When the N-terminal domains of AaApnO and KARI are superimposed, a conformational difference corresponding to an approximately 10° opening of the C-terminal domain of AaApnO relative to the N-terminal domain is observed (Fig. 3*D*). Consequently, in the dimeric structure, the distance between the two C-terminal domains of AaApnO is greater than that in KARI.

### Coenzyme binding mode of AaApnO

Despite no addition of coenzyme during purification and crystallization, clear electron density for the NAD^+^ molecule was fortunately observed in the *F*_o_-*F*_c_ omit map (Fig. 3*E*). It bound at the C-terminal edge of the five-stranded parallel β-sheet (β2-β1-β3-β4-β5) of the N-terminal domain in an extended conformation (Fig. 3*B*). Based on this insight, we measured the absorption spectrum of the purified enzyme, in which an absorption peak at ∼340 nm was found (Fig. 3*F*). Therefore, we assumed that the purified AaApnO contains NAD^+^ (and partially NADH), derived from *Escherichia coli* cells; thus, no addition of coenzyme was necessary for crystallizing the other complex structures. Unless otherwise specified, NAD^+^ is simply omitted from the descriptions of the crystal structure(s) of AaApnO in complex with ligand(s) in the text.

The region of Ala9-Gly10-Gly11-Lys12-Met13-Gly14, located between the end of β1 and the start of α1, corresponded to the consensus motif for coordinating the nucleotide diphosphate group in the Rossmann fold: [Gly/Ala]-X-Gly-X_2_-Gly (orange in Fig. 3*B*) (see Fig. 7*E*). The adenine ring of NAD^+^ binds in a hydrophobic pocket on the enzyme surface formed by the side chains of Phe7, Val31, Pro33, Val66, Pro67, Leu70, and Val74 (Fig. 3*E*). A pyrophosphate moiety interacts with the main and side chains of Lys12 and the main chain of Met13, and the nicotinamide riboside forms a hydrogen bond with the side chain of Asp68.

**Figure 6:**
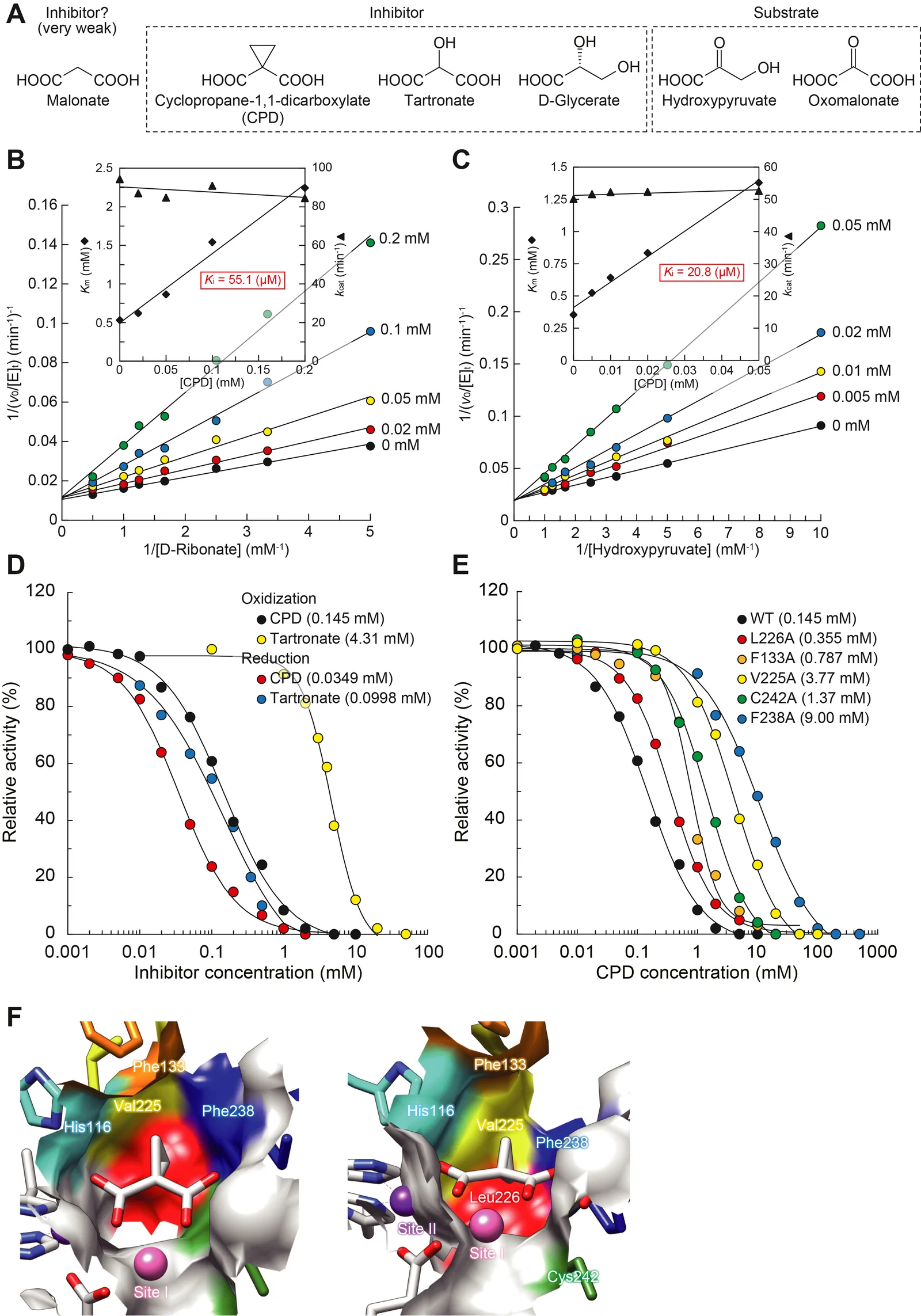
**Inhibition study of AaApnO.***A*, chemical structure of derivatives of malonate. Lineweaver-Burk plots showing competitive inhibition by CPD in the oxidative (*B*) and reductive (*C*) directions. D-Ribonate and hydroxypyruvate concentrations were changed with fixed concentrations of CPD, which are indicated. The inset shows *K*_m_ and *k*_cat_ values *versus* [CPD]. Inhibition experiments with tartronate and glycerate are shown in Fig. S8. *D*, determination of IC_50_ values (in parentheses) by CPD and tartronate. Inhibitor concentrations were changed with a fixed concentration of substrate. Relative specific activity values were expressed as percentages of the values obtained in the absence of inhibitor. IC_50_ values were calculated by curve fitting using ImageJ software (https://imagej.net/ij/). *E*, comparison of IC_50_ values (in parentheses) for CPD between the WT and mutant enzymes. The selected residues form a hydrophobic binding pocket (*F*). ApnO, D-Apionate oxidoisomerase; CPD, cyclopropane-1,1-dicarboxylatel.

**Figure 7:**
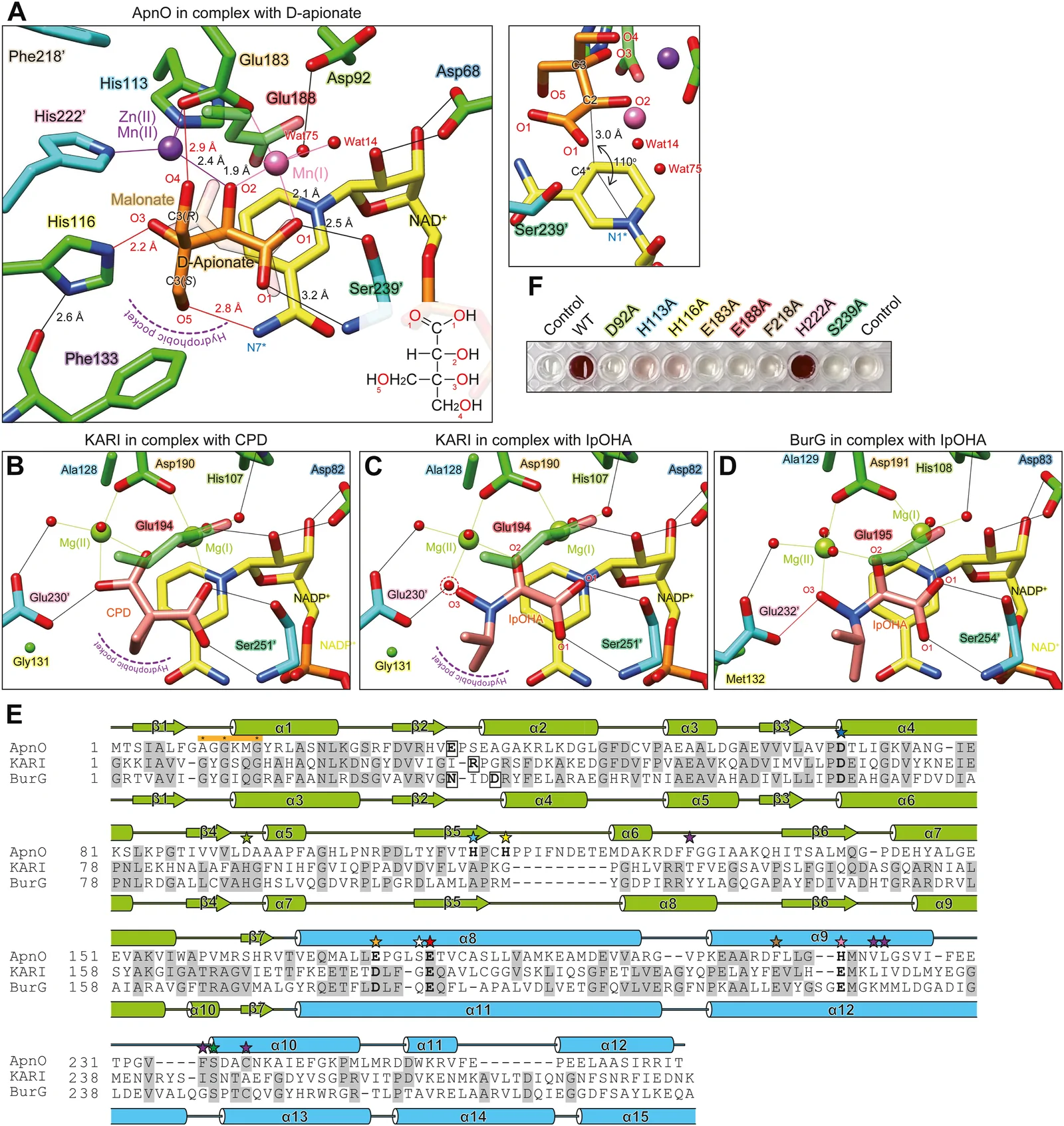
**Substrate binding mode of ApnO.***A*, binding model of D-apionate (inset). *Red*-colored atomic contacts are specifically observed in this model, compared with those of (translucent) malonate. The colors of amino acid residues correspond to the stars in *E*. The *right panel* indicates the relative position between nicotinamide ring and D-apionate. Crystal structures of SaKARI in complex with CPD (5W3K) (*B*) or IpOHA (6AQJ) (*C*). *D*, crystal structure of BurG in complex with IpOHA (7PCL). *E*, structure-based amino acid sequence alignment of AaApnO, SaKARI, and BurG. Conserved residues are shown as gray-shaded letters. The secondary structural assignments for AaApnO and SaKARI, including helices (*cylinders*) and strands (*arrows*), are shown above and below the alignment, respectively. The N- and C-terminal domains are colored in *green and cyan*, respectively. Amino acid residues in *boxes* are responsible for recognizing the 2′-hydroxyl and phosphate groups of ribose in NAD^+^ (for AaApnO and BurG) and NADPH (for SaKARI), respectively. Amino acid residues with stars were selected for site-directed mutagenesis. The *orange-colored* region indicates the consensus motif of the Rossmann fold (Fig. 3*B*). ApnO, D-Apionate oxidoisomerase; CPD, cyclopropane-1,1-dicarboxylatel.

With regard to coenzyme specificity, the 2′- and 3′-hydroxyl groups of NAD^+^ form double hydrogen bonds with the side chain of Glu32 in a loop region between β2 and α2 (Fig. 3*E*), which is completely conserved among other ApnO enzymes. The structurally homologous loop region in KARI also contains a key residue that is responsible for distinguishing between coenzymes: an arginine residue in mostly NADPH-dependent KARI, and a glutamate residue in uniquely NADH-dependent KARI (4XDY; Table S1) and BurG (7PCL) (see Fig. 7*E*).

### Anomalous dispersion analysis of zinc in AaApnO

The active site pocket in “Class I” KARI is formed by regions of the N- and C-terminal domains of one subunit and the C-terminal domain of the second subunit, and “Class II” KARI functions solely as a monomer due to a duplication of the C-terminal domain of Class I 18, 19, 20; two types of KARI form each separate phylogenetic clade (Fig. 3*C*). In this regard, ApnO possesses a topological homology to Class I KARI (21, 22, 23, 24, 25, 26, 27, 28), among which the enzyme from *Staphylococcus aureus* (SaKARI) was used as a reference (24).

Superposition to the crystal structure with that of SaKARI showed that Glu183 and Glu188 in AaApnO are homologous to Asp190 and Glu194 in SaKARI, which coordinate the two magnesium ions, Mg(I) and Mg(II), at sites I and II, respectively. In the putative active site, one sphere of electron density, probably derived from a metal ion, was present at the position almost equivalent to Mg(II) in KARI (Fig. 4*A*). Interestingly, this unidentified (metal) ion is tetrahedrally coordinated by His113, Glu183, His222′, and wat53; a prime indicates a residue in another subunit (Fig. 5*A*). This type of coordination geometry is characteristic of Zn^2+^, whereas Mg^2+^ typically exhibits octahedral coordination with six ligands. Therefore, we performed an X-ray diffraction experiment using AaApnO at the *K*-edge of zinc (a wavelength of 1.28 Å) (26JC in Table 3). In the anomalous difference Fourier map, a clear peak was observed at the expected site II position (orange mesh in Fig. 4*A*) (referred to as Zn(II)). Gerlt and coworkers previously did not discuss the possibility of the presence of Zn^2+^ in ApnO (5). On the other hand, strong negative density was observed in the electron density maps after fitting more than 50% Zn^2+^ occupancy at site II (red mesh in Fig. 4*D*), and re-refinement optimized its occupancy to be 43%. It was impossible to directly determine the zinc content of the purified enzyme by atomic absorption spectrometry analysis, because of unfavorable aggregation during dialysis against water.

Although most crystal structures of KARI contain both Mg(I) and Mg(II) in the active sites, there are two examples in which only Mg(II) is contained; 3FR8 (only in subunit A) (23) and 5YEQ (25) (Table S1); no significant discussion of this feature was provided in either structure.

### Binuclear metal center of AaApnO

As described above, the purified AaApnO showed no activity and required several metal ions. Therefore, to obtain the functional holo-form, AaApnO was alternatively purified and crystallized under a newly identified condition (Index H3) in the presence of Mn^2+^ because of the low reproducibility of crystallization under Index G7.

X-ray diffraction data collected at a wavelength of 1.28 Å (26TF in Table 3) reconfirmed the presence of Zn^2+^ at site II, although the anomalous dispersion peak was weak, compared with the Zn(II) bound form (Fig. 4*B*). Next, X-ray diffraction data were collected at the *K*-edge of manganese (a wavelength of 1.89 Å) (44CF in Table 3). It is likely that collecting these diffraction data was difficult due to the low energy range (6.54 keV), compared with that for zinc (9.66 keV); there is only one other study on protein crystals from our group reported in 2002 (29, 30). Surprisingly, two anomalous dispersion peaks were clearly observed at not only the expected site I but also site II (referred to as Mn(I) and Mn(II), respectively) (purple mesh in Fig. 4*C*). Collectively, these results suggested that the binuclear metal center of ApnO consists of either Zn(II) and Mn(I) or Mn(II) and Mn(I) (referred to as Zn(II)/Mn(II) and Mn(I)); the distance between the two metal ions (3.7 Å) was the same as that between Mg(II) and Mg(I) in SaKARI (Fig. 5*C*). Based on these insights, we again collected X-ray diffraction data of the purified enzyme at a wavelength of 1.89 Å (for Mn^2+^), and found that there was no anomalous dispersion peak at site II (data not shown). This observation provided important evidence that among several (trace) metal ions in the culture medium, Zn^2+^ “selectively” bound to the protein during expression in *E. coli* cells, which must reflect a genuine feature (property) of ApnO, but was not artificially introduced by the purification and crystallization procedures.

Strong negative electron density was observed after fitting only Zn^2+^ at site II, similar to the structure of 26JC (red mesh in Fig. 4*E*). This phenomenon was clearly improved as the ratio of Mn^2+^ was increased, and the respective occupancy rates of Zn^2+^ and Mn^2+^ were optimized to 12% and 88%, respectively, by re-refinement, conforming to the much larger anomalous dispersion peak of Mn^2+^, compared with Zn^2+^ (Fig. 4, *B* and *C*). When compared with the structure of 26JC (43%), the low occupancy of Zn^2+^ may be due to its being (easily and/or gradually) replaced by Mn^2+^ during purification and crystallization (see “Experimental procedures”), which raises one question of whether both metal ions in the binuclear metal center are indeed functional in catalysis. It is noteworthy that since Mg^2+^ is successfully coordinated only at site I, the activity in the presence of Mg^2+^ is derived from the binuclear metal center of Zn(II) and Mg(I). In this regard, it is likely that the binuclear metal center of Zn(II) and Mn(I) is also functionally active, and the binuclear metal center of Mn(II) and Mn(I) is rather artificial.

There is one example of a heterometic binuclear metal center in KARI, which consists of Fe^3+^ and Mg^2+^ fully occupied at sites I and II, respectively (21) (4XIY in Table S1). Since this enzyme is serendipitously crystalized in absence of metal ion, there is no report whether the binuclear metal center is functional nor not.

### Binding mode of malonate

In the Zn(II)/Mn(II) and Mn(I) bound form of AaApnO (26TF and 44CF in Table 3), clear electron density in the *F*_o_–*F*_c_ omit map also allowed the modeling of malonate (Fig. 5*G*), probably derived from the crystallization buffer (tacsimate). There is one crystal structure of KARI in complex with malonate (7TOC in Table S1), which was also crystallized in the presence of 2 M malonate. Although no inhibition by malonate (at least up to 100 mM) was observed for AaApnO (data not shown), a more concentrated solution may affect the activity. In fact, despite the extremely high *K*_i_ value (2.5 M), ethylene glycol, derived from the crystallization buffer, binds to the crystal structure of KARI (23) (3FR8 in Table S1).

Malonate is a prochiral molecule with two carboxyl groups. The C1 carboxyl group interacts with Mn(I) and the side chain and the main chain nitrogen atom of Ser239′, and the C3 carboxyl group with Zn(II)/Mn(II), Mn(I), and the side chain of His116. With regard to metal ion binding, Zn(II)/Mn(II) is tetrahedrally coordinated by His113, Glu183, His222′, and the O3A atom of malonate, as in the Zn(II) bound form (Fig. 5*C*). This is unexpected because this type of coordination geometry for Mn^2+^ has been reported only for superoxide dismutase 2 (31). Mn(I) is typically octahedrally coordinated with Glu183, Glu188, the O1A and O3A atoms of malonate, wat14, and wat75. Furthermore, there are hydrogen bonds between wat75 and Asp92, and between Glu183 and Ser187. Among these active site residues, all amino acid residues except Ser187 are completely conserved among other ApnO enzymes.

We also analyzed the crystals obtained under the same crystallization condition in the presence of Ca^2+^ instead of Mn^2+^ (43IA in Table 3). Although no calcium ion was bound in this structure, the C1-C2-C3 backbone of malonate was slightly closer to the nicotinamide ring, and wat2 (and wat2) was located at the equivalent positions of Mn(I) (and wat75) (Fig. 5, *D* and *H*), suggesting the possibility that a metal ion at site I is not directly necessary for substrate binding.

Surprisingly, in other crystals obtained under the same crystallization condition, continuous electron density between the C2 atom of malonate and the C4∗ atom of the nicotinamide ring of NAD^+^ was observed, and the distance of 1.5 Å corresponded to a covalent bond (Fig. 5, *E* and *I*). In this structure (26TG in Table 3), the C1-C2-C3 backbone of malonate is rotated by 180° relative to that of the non-covalently bound form. Indeed, we also found that only the electron densities corresponding to two the carboxyl groups of malonate were visualized (Fig. S7), suggesting the exchange of malonate with two different forms.

To our best knowledge, a similar modification of the nicotinamide ring is known in only two proteins (see Fig. 8*C*). First, isoniazid (a prodrug) is activated to an isonicotinic acyl radical by KatG from *Mycobacterium tuberculosis*, which spontaneously couples with NADH at the C4∗ position. The resultant nicotinoyl-NADH adduct finally inhibits InhA involved in the synthesis of the mycobacterial cell wall (32). Second, when homoserine dehydrogenase from archaea is crystallized in the presence of concentrated cysteine (a competitive inhibitor), the formation of a covalent bond between the sulfur atom of cysteine and the C4∗ atom of NAD^+^ is observed in the crystals (33). Among them, this crystal structure of AaApnO is similar to the latter, and is significantly helpful for elucidating the catalytic mechanism (see below).

**Figure 8:**
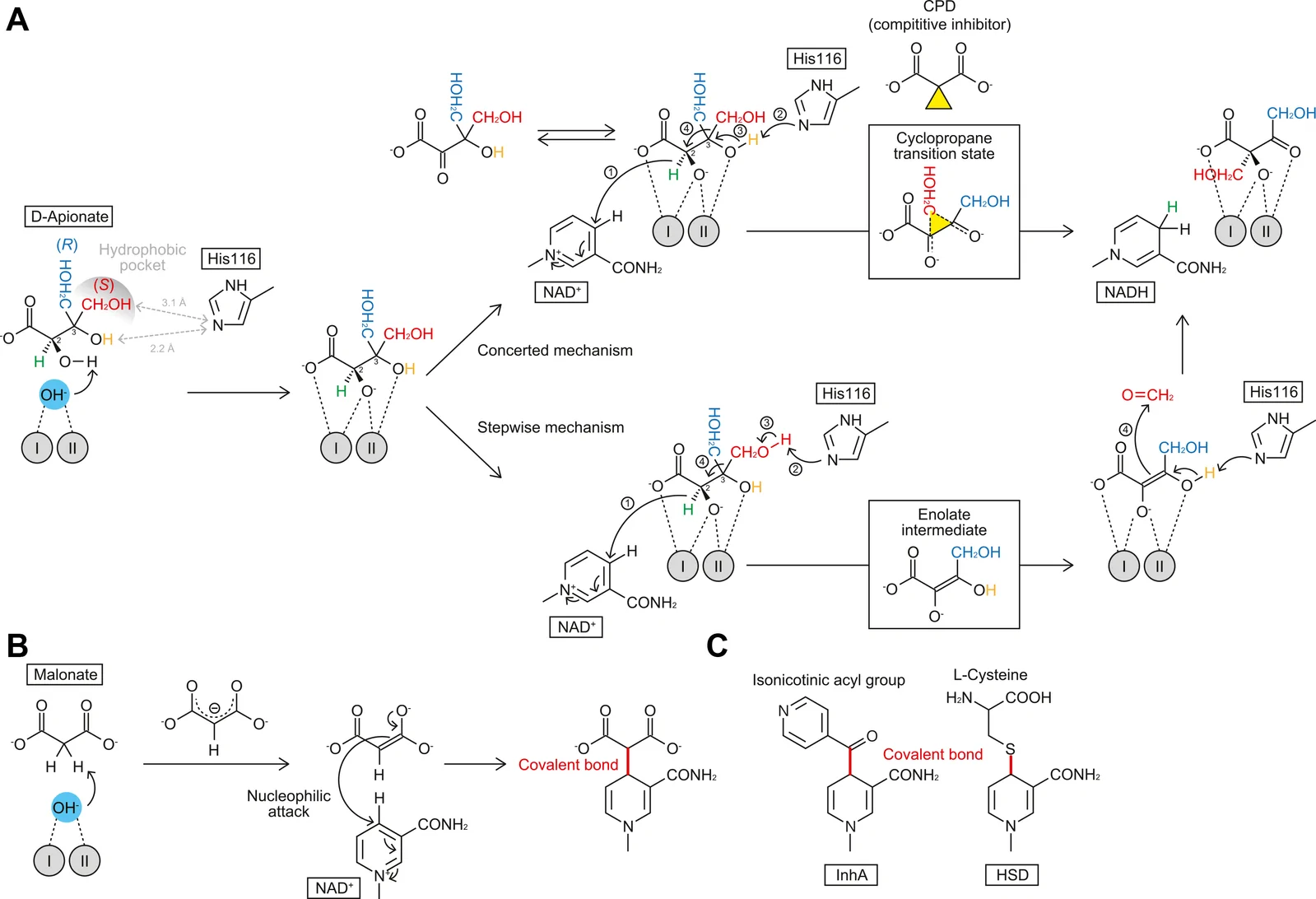
**Proposed reaction mechanism of ApnO.***A*, reaction mechanism for D-apionate as a substrate, with concerted and stepwise routes. *B*, hypothetical mechanism for the formation of a covalent bond (*red*) between malonate and NAD^+^ in crystals. *C*, covalent modification at C4∗ atom of nicotinamide ring in InhA (*left*) and homoserine dehydrogenase (*right*). ApnO, D-Apionate oxidoisomerase.

### Identification and binding mode of inhibitor

We attempted to identify derivative(s) of malonate that function efficiently as inhibitors (Fig. 6*A*). Among the tested compounds, cyclopropane-1,1-dicarboxylate (CPD) competitively inhibited both the oxidation and reduction activities, with *K*_i_ values 13- and 24-fold lower than the *K*_m_ values for D-ribonate and hydroxypyruvate; 55.1 μM and 20.8 μM, respectively (Fig. 6, *B* and *C*). Alternatively, the CPD concentrations were changed with the fixed concentration of substrate, from which the IC_50_ values were determined to be 0.145 mM and 0.0349 mM, respectively (Fig. 6*D*). Competitive inhibition in the reductive reaction was also observed with tartronate and D-glycerate, whereas their *K*_i_ values were 6.1- and 22-fold higher than that of CPD; 127 μM and 464 μM, respectively (Fig. S8).

Based on these results, AaApnO was co-crystallized with CPD under a newly identified condition (Index F2), in which malonate was not contained (26TH in Table 3). Clear density in the *F*_o_–*F*_c_ omit map allowed the modeling of CPD (Fig. 5*J*). The malonate moiety of CPD is slightly rotated by approximately 17°, and the cyclopropane ring is accommodated within a binding pocket with an apolar region composed of the side chains of Phe133, Val225, Leu226, Phe238, and Cys242 (Fig. 5*F*). Collectively, these results suggested that the binding mode of CPD was highly similar to that of malonate, and that the cyclopropane ring plays an important role in its strong inhibitory activity, probably by mimicking the transition state (see below).

### Binuclear metal center without malonate and inhibitor

To elucidate the nature of the binuclear metal center before and after substrate binding in more detail, we further determined the crystal structure of the ligand-free form (26MW in Table 3). When compared with the malonate (or CPD) bound form, the O3A, O3B, and O1A atoms of malonate are replaced by wat48, wat171, and wat664, respectively (Fig. 5*B*). Two water molecules, wat168 and wat299, are located at the equivalent positions of wat14 and wat75, respectively. Importantly, wat48, which bridges the binuclear metal center, is farther from Mn(I) than the O3A atom of malonate (the distance increases from 2.3 Å to 2.8 Å), making the tetrahedral and octahedral coordination geometries more distorted. In other words, it is likely that substrate binding favorably stabilizes the binuclear metal center for hydride transfer to NAD^+^ (see below).

### Model of substrate binding

To elucidate the structural basis of substrate specificity in more detail, we constructed a model structure in complex with D-apionate using the NAD^+^, Zn(II)/Mn(II), Mn(I), and malonate bound form (Fig. 7*A*). When compared with bound malonate, the C1-C2-C3 backbone of D-apionate was rotated relative to the two metal centers by approximately 180°, such that the O2 and O3 atoms were overlaid with the O3A and O3B atoms of malonate, respectively. A similar phenomenon is also observed between the crystal structures of SaKARI in complex with CPD or IpOHA (Fig. 7, *B* and *C*) (24). IpOHA (*N*-isopropyloxalyl hydroxamate) is a competitive inhibitor that mimics the transition state of the 2-oxo acid intermediate (see Fig. S10*B*). The C2(*R*) hydroxyl group of D-apionate was favorably positioned with respect to the hydride-accepting NAD^+^; the distance between the C2 atom of malonate and the C4∗ atom of the nicotinamide ring of NAD^+^ and the angle defined by N1∗-C4∗-C2 were 3.0 Å and 110°, respectively. The distance between the C3 hydroxyl group and His116 was consistent with hydrogen bond formation, thereby enabling His116 to abstract a proton as a general base catalyst (see below).

The carbon backbone of modeled D-apionate corresponded to a C1-C2-C3 framework bearing two C3 hydroxymethyl groups, rather than a C1-C2-C3-C4 framework bearing one C3 hydroxymethyl group. Among them, the C3(*R*)-group is close to the side chain of Glu183. On the other hand, the C3(*S*)-group is accommodated within the same hydrophobic pocket as the cyclopropane ring of CPD, and its hydroxyl group interacts with the N7∗ atom of NAD^+^ (at a distance of 2.8 Å). No homologous interaction corresponding to the latter is found in the model structures in complex with D-erythronate or D-ribonate (Fig. S9), which may explain why D-apionate shows relatively low *K*_m_ values, compared with the other substrates (Table 1). Importantly, as described above, the reaction product of AaApnO was not identified, and there remained another possibility that ApnO directly oxidizes the substrate at the C3 position. In this regard, this model structure provided strong evidence that oxidation by ApnO must occur at the C2 hydroxyl group.

### Site-directed mutagenic study

Based on these structural insights, several (putative) active site residues were substituted with alanine; Asp68, Asp92, His113, His116, Phe133, Glu183, Ser187, Glu188, Phe218, His222, Val225, Leu226, Phe238, Ser239, and Cys242 (residues marked with stars in Fig. 7*E*).

Mutants of the completely conserved residues among ApnO enzymes (underline) were mostly inactive or showed dramatically decreased activity (Table 4), consistent with their direct interactions with the binuclear metal center (His113, Glu183, and Glu188), coenzyme (Asp68), and substrate (His116 and Ser239) (Fig. 7*A*). Similar results were obtained in the zymogram staining analysis (Fig. 7*F*). In this regard, it is unexpected that despite its coordination to Zn(II)/Mn(II), the H222A mutation had no clear effect on catalysis. There is one possibility that the “plasticity” at this metal ion binding site enables replacement of His222 by a water molecule without loss of the precise coordination geometry. A similar phenomenon has been reported in another protein (34). Asp92 may play a role in favorably positioning wat51, which ligates to Mn(I). Among Asp68, Asp92, and Ser239, which are close to the nicotinamide moiety of NAD^+^, the absorption peak at ∼340 nm was eliminated in the purified D68A and S239A mutants, unlike the WT enzyme (Fig. 3*F*), suggesting the release of the coenzyme, probably caused by weakened interaction with the protein.

**Table 4:** Kinetic parameters for D-ribonate by mutants of AaApnO

Enzyme	K_m_ (mM)	k_cat_ (min^−1^)	k_cat_/K_m_ (min^−1^ mM^−1^)
WT	0.743 ± 0.040^a^	203 ± 5	274 ± 7
D68A	N.D.^b^	N.D.	N.D.
D92A	N.D.	N.D.	N.D.
H113A	0.475 ± 0.082^a^	0.0121 ± 0.0008	0.0258 ± 0.003
H116A	N.D.	N.D.	N.D.
F133A	0.0917 ± 0.0061^a^	5.12 ± 0.05	56.0 ± 3.0
E183A	N.D.	N.D.	N.D.
S187A	0.0920 ± 0.006^a^	5.26 ± 0.05	57.5 ± 3.1
E188A	N.D.	N.D.	N.D.
F218A	N.D.	N.D.	N.D.
H222A	0.116 ± 0.005	36.8 ± 1.2	318 ± 2
V225A	0.856 ± 0.043^a^	145 ± 4	169 ± 4
L226A	0.576 ± 0.080^a^	37.5 ± 2.9	65.4 ± 3.7
F238A	0.301 ± 0.005^a^	11.8 ± 0.1	39.3 ± 0.5
S239A	N.D.	N.D.	N.D.
C242A	0.448 ± 0.064^a^	49.3 ± 3.3	111 ± 8

Values are the means ± SD, *n* = 3.

a Eight different substrate concentrations between 1 and 10 mM were used.

b Not assessed due to extremely weak activity. ApnO, D-Apionate oxidoisomerase.

The F133A, V225A, L226A, F238A, and C242A mutants, involved in the formation of the hydrophobic pocket for substrate binding (Fig. 6*F*), showed significant activity with moderately decreased *k*_cat_ values (Table 4), whereas they commonly showed decreased IC_50_ values for CPD, compared with the WT enzyme, particularly the F238A and V225A mutants (62- and 26-fold, respectively) (Fig. 6*E*). Importantly, since they were assayed using D-ribonate as a substrate, there is one possibility that more critical effect is found in the activity for D-apionate. The homologous mutations in KARI result in complete loss of activity or a dramatic decrease in activity (27).

### Catalytic mechanism by ApnO

By analogy to the initial step in the catalytic cycles of KARI (24), a hydroxide ion deprotonates the C2 hydroxyl group of D-apionate to form the corresponding alkoxide (Fig. 8*A*). Although no hydroxide is directly observed in most crystal structures of KARIs, the Zn(II)/Mn(II) and Mn(I) bound form of AaApnO, determined in this study, clearly contains a water molecule (wat48) that bridges the binuclear metal center as a candidate (Fig. 5*B*), similar to BurG (15). Formation of a complex between the alkoxide and the binuclear metal center enables favorable positioning of the substrate above the nicotinamide ring of NAD^+^ for hydride transfer. Furthermore, the protonated state of the ND1 atom of His116 is likely stabilized through interaction with the main-chain carbonyl oxygen and the hydrophobic environment created by the bulky side chain of Phe133 (Fig. 7*A*).

This hypothetical scheme can also explain why the covalent modification between NAD^+^ and malonate is unusually observed (Fig. 5, *E* and *I*). Since according to the Bordwell p*K*_a_ scale in DMSO (35), malonate (and its esters) exhibits much higher C-H acidity than simple methylene compounds such as alkanes and ethers, the hydroxide ion enables direct abstraction of one of the C2 protons (Fig. 8*B*). For this purpose, the C1-C2-C3 backbone of malonate must be absolutely located at the equivalent position to that of D-apionate. Although the non-covalently bound form is not resolved, the visualization of the two carboxyl groups of malonate may partially support it (Fig. S7). The resulting enolate is conjugated and thus resonance-stabilized, and then undergoes nucleophilic attack at the C4∗ atom of the nicotinamide ring of NAD^+^, resulting in covalent bond formation between malonate and NAD^+^. On the other hand, this structure may be due to the artificial conditions of concentrated malonate, and may have no physiological significance.

There are two possible mechanisms for the second step of the catalytic reaction by ApnO (Fig. 8*A*). First, the concerted mechanism proceeds through hydride transfer from the C2 to NAD^+^ (1), deprotonation of the C3 hydroxyl group by His116 as a general base catalyst (2), formation of a 3-oxo group (3), and migration of 3(*S*)-hydroxymethyl group to the C2 position (4). The polarization of both the C2 and C3 carbonyl groups by the binuclear metal center enhances the isomerization. The O7∗ atom of NADH potentially forms a hydrogen bond with the 3(*S*)-hydroxymethyl group, which enables the migration of the hydrophilic branched group within the non-polar pocket (Fig. 7*A*); the migration of the relatively small hydrogen atom in other substrates is easier (Fig. S3). His116 is immediately deprotonated at the optimum pH (∼10) of AaApnO. The alternative stepwise mechanism consists of hydride transfer from C2 to NAD^+^ (1), deprotonation of the hydroxyl group of one of two C3 hydroxyl methyl groups by His116 (2), and release of a formaldehyde group (3), followed by the formation of an enolate intermediate. Finally, nucleophilic attack on the C2 position results in the formation of the C2 hydroxyl methyl group (4). Among the two hypothetical mechanisms, the former is favorably supported by the short distance between His116 and its target chemical groups (2.2 Å and 3.1 Å), and by competitive inhibition by CPD, which mimics the cyclopropane transition state.

## Discussion

More than 40 types of branched sugars, including D-apiose, have been identified in nature as the building blocks of glycosides, nucleosides, and antibiotics: L-streptose is well known to be as the monosaccharide skeleton of streptomycin. Although there is no significant insight into the (microbial) degradation of these sugars, the most interesting (and important) step may be how to remove or linearize their branching groups. In the case of D-apiose, this problem is mainly resolved by C1 decarboxylation of 3-oxo-D-isoapionate (or 3-oxo-D-isoapionate 4P) (Fig. 1*A*), rather than by the direct removal of the C3 hydroxylmethyl group.

### Molecular evolutional insight between ApnO and KARI

Despite the similar reaction, the (structure-based) sequence identity between ApnO and KARI (∼15%) is not particularly high, compared with other structural homologs (Fig. 3*C*). On the other hand, there are four active site residues that are structurally conserved between them, which commonly play roles in directly interacting with the nicotinamide ribose of the coenzyme (blue), metal ions (red), or the C1 carboxyl group of the substrate (green) (Fig. 7*E*). It is noteworthy that all of them are located around metal ion binding site I with the same octahedral coordination geometry, strongly indicating a common evolutionary origin.

Hertweck and coworkers proposed that the reaction of “canonical” KARI, which proceeds through the cyclopropane transition state, was repurposed as that of BurG in the biosynthesis of polyketides (malleicyprols) by pathogenic *Burkholderia pseudomallei* (15). Since most active-sites are completely conserved (Fig. 7*E*), this evolutionary trajectory is reasonable. On the other hand, SaKARI and BurG possess the (completely conserved) glutamate residue at the sequentially equivalent position to His222 in AaApnO (pink); Glu230 and Glu232, respectively. BurG utilizes Glu232 as a general base that deprotonates at the C3 position of gonydiol during the catalytic reaction (15) (Figs. 7*D* and S10*A*). On the other hand, when KARI is crystallized in the presence of IpOHA and NADPH, the hydride attached to the C4 carbon of the nicotinamide moiety protonates the nitrogen of IpOHA (to yield deoxy-IpOHA), resulting in the release of the hydroxyl group, which may become protonated by Glu230, a ligand for Mg(II) (24) (Figs. 7*C* and S10*B*). Their catalytic roles are likely similar to that of His116 in AaApnO (yellow) (but not His222), and the misalignment between sequence and function is due to the relative positioning between the N- and C-terminal domains (Fig. 3*D*).

As described above, the H222A mutant of AaApnO shows similar activity, compared with the WT enzyme (Table 4). Indeed, in the zymogram staining analysis performed for ∼1 h, trace activity was observed in the H113A and H116A mutants (but not in the spectrophotometric assay) (Fig. 7*F*); Mn(I) may be fundamentally enough for the catalysis. Collectively, these indicate that ApnO and KARI/BurG had independently acquired metal ion binding site II and their functional basic residues during the evolutionary process.

### Metal ion selectivity of ApnO

If, based on these reaction schemes, the same binuclear metal center is involved in both the oxidative and (reverse) reductive reactions, why dose AaApnO uniquely show different metal ion selectivity between them (Table 2)? We assume that, in the oxidative reaction, the binding of Mn^2+^ and Mg^2+^ at site I (but not Ni^2+^ or Co^2+^) enables the formation of a hydroxide from the water molecule that coordinates to Zn(II) (Fig. 5, *A* and *B*). Generally, whether water bound to a metal ion (probably wat53 bound to Zn(II)) exists as a hydroxide depends on the charge density of the metal ion, the coordination number, and/or surrounding ligands, as described in carbonic anhydrase, carboxypeptidase, and metallo-β-lactamase (36). In the reductive reaction, the binuclear metal center may play a role in simply positioning the substrate (in particular, the 2-oxo group), which is also sufficient for Ni^2+^ and Co^2+^, because they have the same octahedral coordination geometry as Mn^2+^ and Mg^2+^.

Similar differences in kinetic behavior depending on metal ion are also found in KARI. Mg^2+^ is competent in both the isomerization and reduction (also reverse oxidization) reactions, whereas Mn^2+^ only catalyzes the reduction reaction (but not Ni^2+^ and Co^2+^). Although in one crystal structure of KARI in complex with two Mn^2+^ ions (1QMG in Table S1), the nature of the binuclear metal center and its neighboring residues is almost the same as those observed for Mg^2+^ (19), it is proposed that the isomerization reaction (but not reduction) requires a very precise orientation of the 3-oxo acid substrate with respect to the metal ion (4), which partially overlaps with our hypothesis. Furthermore, in case of ApnO, it is noteworthy that, among the tested metal ions, only Mn^2+^ can precisely (fully) coordinate to both metal ion binding sites I and II, which may also be responsible for its significant preference for Mn^2+^ over Mg^2+^.

### Metabolic linkage of ApnO

ApnO-OiaK-OiaT genes, related to route 2 of the D-apionate pathway, are located adjacent to the C4/C5/C6 sugar acid gene cluster for the non-phosphorylating pathways of C6 (D-gluconate and D-galactonate), C5 (D- and L-arabinonate and D-xylonate), and C4 (L-threonate and D-erythronate) sugar acids, but not D-ribonate (Fig. 1*D*). D-Ribonate is an intermediate of the non-phosphorylating D-ribose pathway from archaea (Fig. S11). Therefore, this genome context enables the metabolism of D-ribonate through the C4 sugar acid pathway, which provides an evolutionary advantage for bacteria to utilize otherwise no-metabolizable carboxylates (Fig. 1*J*); its physiological role is similar to that of Ltn3D.

On the other hand, ApnO-OiaC-LerK-EryH-EryI genes, related to route 3 of the D-apionate pathway, are closely located with a dehydrogenase (Rib2D) and an isomerase (OrnI), involved in D-ribonate metabolism (11) (Fig. 1*E*). Indeed, the EryH-EryI genes are (frequency) separately located from ApnO-OiaC-LerK, as observed in *A*. *altamirensis* (Fig. 1*G*), and are alternatively clustered with the EryA-EryB-EryC genes related to erythritol metabolism (37) (Fig. 1*A*). Since LerK can phosphorylate not only L-erythrulose but also D-erythrulose (5), this genome context may also serve as a “metabolic funnel” toward D-erythrose 4P.

### Molecular evolution of D-apiose and D-apionate pathways

The origin of D-apionate in nature is mainly attributed to the oxidization and subsequent hydration of D-apiose by D-apiose dehydrogenase and D-apionolactonase. Therefore, it may be unusual that routes 2 and 3 start from D-apionate, compared with route 1 (Fig. 1*A*). Indeed, we found that on many bacterial genomes, metabolic genes, related to routes 2 and 3, are clustered with D-apionolactonase and putative dehydrogenase/reductase genes (Fig. 1, *H* and *I*). This type of genome context had not previously been discovered by Gerlt and coworkers (5), and we are currently working to functionally characterize the latter as a novel type of D-apiose dehydrogenase. In this regard, further bioinformatic and biochemical analyses may provide evidence for convergent evolution of other D-apiose/D-apionate metabolic enzymes including ApnO.

## Experimental procedures

### General procedures

*A. altamirensis* NBRC 107774 was purchased from the National Institute of Technology and Evaluation. Bacterial genomic DNA was prepared using a DNeasy Tissue Kit (Qiagen). PCR was performed using a GeneAmp PCR System 2700 (Applied Biosystems) for 30 cycles in a 50 μl reaction mixture containing 1 unit of KOD One DNA polymerase (Toyobo), appropriate primers (15 pmol), and template DNA under the following conditions: denaturation at 98 °C for 10 s, annealing at 55 °C for 5 s, and extension at 68 °C for a period calculated based on an extension rate of 1 kbp/sec. C5 and C6 sugar acids were mostly prepared by hypoiodite-in-methanol oxidation from the corresponding monosaccharides as Ba^2+^ salts and purified using a column of AG 1-X8 Resin (200–400 mesh, formate form) (Bio-Rad) 8, 10. D-Erythronate (K^+^ salt) and D-gulonate (in the form of 1,4-D-gulonolactone) were purchased from Combi-Blocks and MedChemExpress, respectively.

### Expression and purification of recombinant proteins

The primer sequences used in the present study are shown in Table S2. The ApnO gene of *A. altamirensis* DSM 21988 (Uniprot ID: A0ABY1INU2) was amplified by PCR from the genomic DNA of *A. altamirensis* NBRC 107774 and was introduced into the *Bam*HI-*Hin*dIII site in pQE-80L (Qiagen), a plasmid vector that encodes an N-terminal (His)_6_-tag fused to the expressed protein, to obtain pQE-ApnO.

*E.* c*oli* DH5α transformed with pQE-ApnO vector was grown at 37 °C in LB medium containing ampicillin (50 mg/l). At an OD_600_ of 0.8, isopropyl-β-D-thiogalactopyranoside was added (to a final concentration of 1 mM), and incubation was continued overnight at 25 °C. Subsequently, the cells were harvested by centrifugation at 8000*g* for 5 min and stored at −35 °C.

The frozen bacterial cell mass was resuspended in Buffer A (50 mM sodium phosphate buffer (pH 8.0) containing 300 mM NaCl and 10 mM imidazole), disrupted by sonification using an Ultra Sonic Disruptor UD-211 (Tomy Seiko), and centrifuged at 15,000*g* for 40 min at 4 °C to pellet the insoluble debris. The supernatant was loaded onto a Ni-NTA Superflow column (Qiagen), equilibrated with Buffer A, and the column was then washed with Buffer B (pH 8.0, Buffer A containing 25 mM imidazole instead of 10 mM imidazole). The enzyme was eluted with Buffer C (pH 8.0, Buffer A containing 250 mM imidazole instead of 10 mM imidazole), and the eluate was loaded onto a HiLoad 16/600 Superdex 200 pg column (GE Healthcare) equilibrated with Buffer D (20 mM Tris-HCl (pH 8.0) containing 150 mM NaCl). If necessary, 1 mM metal ion(s) were added to Buffer D. Single peak fractions were concentrated to ∼20 mg/ml using Amicon Ultra-15 (Millipore) and immediately used for crystallization. Alternatively, the purified AaApnO solution was dialyzed against Buffer E (50 mM Tris-HCl (pH 8.0) containing 50% (v/v) glycerol) and stored at −35 °C until functional characterization.

### Site-directed mutagenesis

Several mutants of AaApnO were constructed by a PCR-based method with forward and reverse primers (Table S2) and the cloned pQE-ApnO plasmid as a template. Mutant proteins were expressed and purified by the same procedures as those used for the WT enzyme.

### Enzyme assay

The oxidative dehydrogenase activity for sugar acids was measured in 50 mM glycine-NaOH buffer (pH 10) containing 1 mM substrate, 1 mM MnCl_2_, and 1.5 mM NAD^+^. The reverse reductase activity was measured in 50 mM Hepes-NaOH buffer (pH 7.2) containing 1 mM substrate, 1 mM MnCl_2_, and 0.15 mM NADH. The consumption or formation of NADH was spectrophotometrically monitored at 30 °C as the decrease or increase in absorbance at 340 nm with an extinction coefficient (*ε*) of 6220 M^−1^ cm^−1^ using a Shimadzu UV-1800 spectrophotometer (Shimadzu GLC Ltd). *K*_m_ and *k*_cat_ (=*V*_max_/[E]_t_) values were calculated by directly fitting the Michaelis–Menten equation, *v*_o_/[E]_t_ = *k*_cat_[S]/(*K*_m_ + [S]), to the primary data plotted as [S] *versus v*_0_/[E]_t_ using GraphPad Prism software (ver. 10.6.1).

For the coupled-enzyme spectrophotometric assay, the initial solution (1 ml) contained 50 mM glycine-NaOH buffer (pH 10), 1.5 mM D-erythronate, 1.5 mM ATP, 1 mM MgCl_2_, and AaApnO (10 μg), together with OtnK (10 μg), OtnC (10 μg), and/or DhaK (10 μg). The reaction was started by the addition of 15 mM NAD^+^ (100 μl), and the absorption at 340 nm increased with the production of NADH. NAD(H)-dependent G3PDH (Oriental Yeast; 1 unit) was subsequently added. OtnK, OtnC, and DhaK were prepared as described previously (10).

Alternatively, the NADH-dependent reductive reaction and continuous reaction(s) through AaApnO, OtnK, and/or OtnC were analyzed at 35 °C on an Aminex HPX-87H Organic Analysis column (300 × 7.8 mm, Bio-Rad) linked to an RID-8020 refractive index detector (Tosoh) and eluted with 5 mM H_2_SO_4_ at a flow rate of 0.6 ml/min.

In the zymogram staining analysis, the purified enzyme was incubated in a staining solution consisting of 50 mM glycine-NaOH (pH 10), 1 mM MnCl_2_, 0.25 mM nitroblue tetrazolium, 0.06 mM phenazine methosulfate, 10 mM D-ribonate, and 10 mM NAD^+^ at room temperature. Dehydrogenase activity appeared as a dark color development in the solution.

### Crystallization and X-ray crystallography

Crystallization experiments of AaApnO were performed using the sitting drop vapor diffusion method at 20 °C. Drops (0.5 μl) of ∼20 mg/ml AaApnO protein in Buffer D were mixed with equal volumes of reservoir solution and equilibrated against 70 μl of the same reservoir solution by vapor diffusion. The initial screening was performed using Index HT and Crystal Screen (Hampton Research), and the conditions of Index G7, H3, and F2 were further optimized. For co-crystallization experiments, CPD and a metal ion were added to the enzyme solution to final concentrations of 1 mM and 10 mM, respectively. The crystals obtained were cryoprotected with reservoir solution supplemented with 30% (w/v) polyethylene glycol 3350 (for Index G7 and H3) or polyethylene glycol monomethyl ether 2000 (for Index F2), and were flash-cooled and kept in a stream of nitrogen gas at 100 K during data collection.

Diffraction data were automatically collected using the ZOO system with a PILATUS 6M detector of BL45XU at SPring-8 (Hyogo, Japan), and processed with XDS (38, 39, 40). The structure of AaApnO was solved by the molecular replacement method using the molecular-replacement pipeline program BALBES (41) with the structure predicted by *AlphaFold2* (13) as the search model. Further model building for all structures was performed manually with COOT (42), and crystallographic refinement was carried out with PHENIX (43). Detailed data collection and processing statistics are shown in Table 3.

### Molecular docking

Molecular docking was performed using the OEDocking package (version 4.3.2.1) (44). The crystal structure of AaApnO in complex with NAD^+^, Zn(II)/Mn(II), Mn(I), and malonate (26TF) was processed with the Make_Receptor module to generate the receptor input. Conformer generation of substrates was performed using OMEGA (version 6.0.0.1) in the “pose” mode (45, 46). The resulting conformers were then docked into the active site using the FRED module 47, 48. During docking, constraints were applied to enforce hydrogen bonding to Ser239′ and coordination to the binuclear metal center.

### Sequence comparison

Protein sequences were analyzed using the Protein-BLAST and Clustal W programs distributed by the Kyoto Encyclopedia of Genes and Genomes (KEGG) (www.kegg.jp/kegg/kegg1.html) (49). An unrooted maximum likelihood-based phylogenetic tree of ApnO and its structural homologs was visualized using TreeView X.

## Data availability

Atomic coordinates and structural factor files have been deposited in the Protein Data Bank under accession codes 43IC, 26JC, 26TF, 44CF, 43IA, 26TG, 26TH, and 26MW.

## Supporting information

This article contains supporting information.

## Conflict of interest

The authors declare that they have no conflicts of interest with the contents of this article.
