# Supplementary material for: Structural basis for oxidation-coupled isomerization by ApnO
Source: J Biol Chem. 2026 Oct 9;302(11):113628. doi: 10.1016/j.jbc.2026.113628 (PMC13753588; doi:10.1016/j.jbc.2026.113628)
Supplement: Supplementary file 1 [file mmc1.pdf]

Supplementary information

Structural basis for oxidation-coupled isomerization by ApnO

Seiya Watanabe[1,2,3#*], Yuzuki Aono[2#], Shin-ichi Terawaki[4], Himika Sato[1], Taiyo Yokoi[1,2]

[1] Department of Bioscience, Graduate School of Agriculture, Ehime University, Matsuyama, Ehime, Japan
[2] Faculty of Agriculture, Ehime University, Matsuyama, Ehime, Japan
[3] Center for Marine Environmental Studies (CMES), Ehime University, Matsuyama, Ehime, Japan
[4] Division of Structure Analysis of Protein Complex, Proteo-Science Center (PROS), Ehime University, Matsuyama, Ehime, Japan

[#] Contributed equally

[*]For correspondence. E-mail: irab@agr.ehime-u.ac.jp; Tel./Fax: +81-89-946-9848

Running title: Crystal structure of D-apionate oxidoisomerase
Keywords: D-apionate oxidoisomerase, gene cluster, anomalous dispersion analysis, binuclear metal center, ketol-acid reductoisomerase

This PDF file includes:
Tables S1-S2
Figures S1 to S11

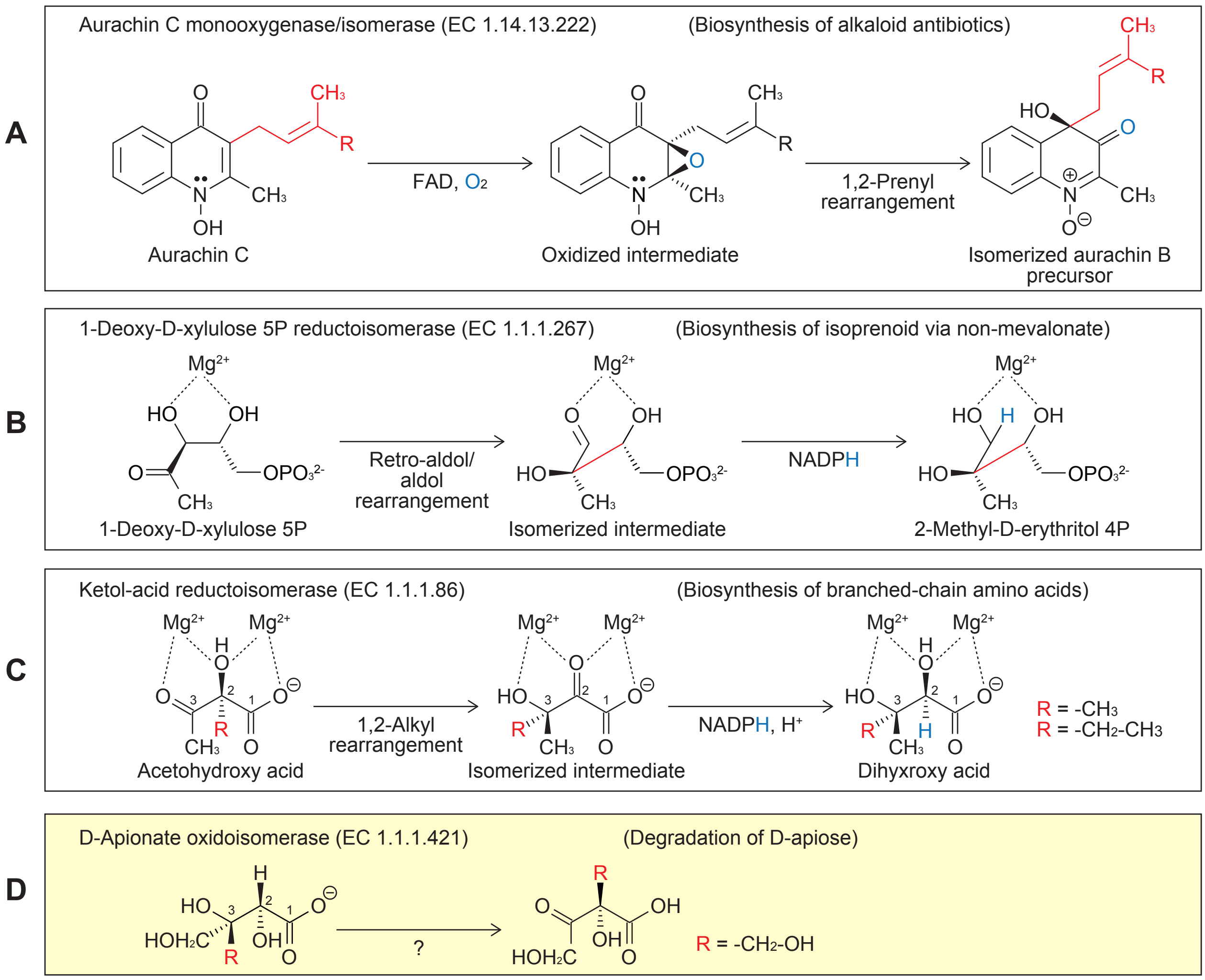

Figure S1. Alkyl (red) migration reactions coupled with oxidoreductase chemistry.
(A) Aurachin C monooxygenase/isomerase (AuaG). (B) 1-Deoxy-D-xylulose 5-phosphate reductoisomerase (DXR). (C) Ketol-acid reductoisomerase (KARI). (D) Reaction of D-apionate oxidoisomerase (ApnO).

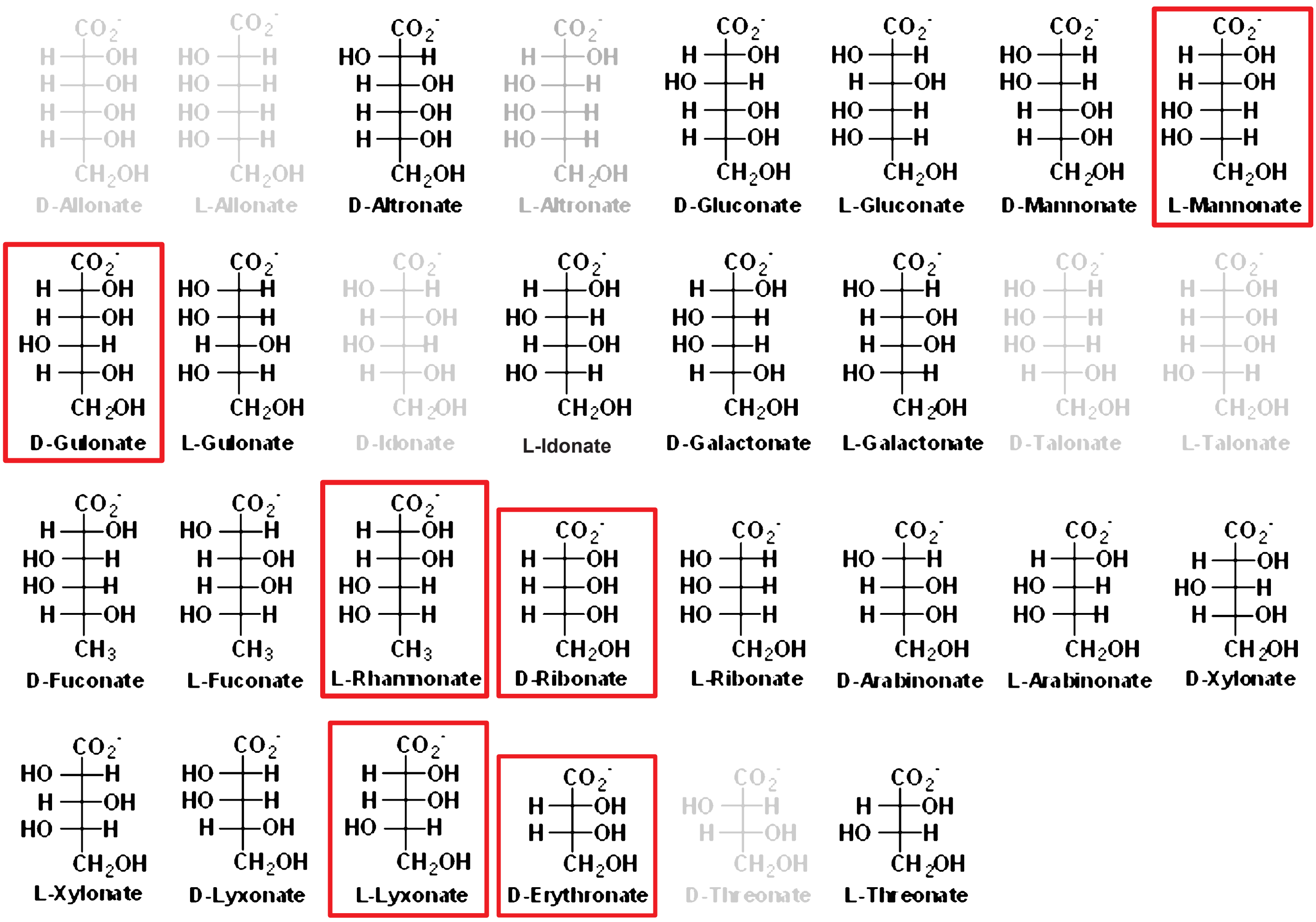

Figure S2. Structural formula of C4/C5/C6 sugar acids.
Among them, compounds except translucence were purchased or chemically syhthesized, and screened as potential substrates for the recombinant AaApnO protein. Compounds in red boxes are active substrates.

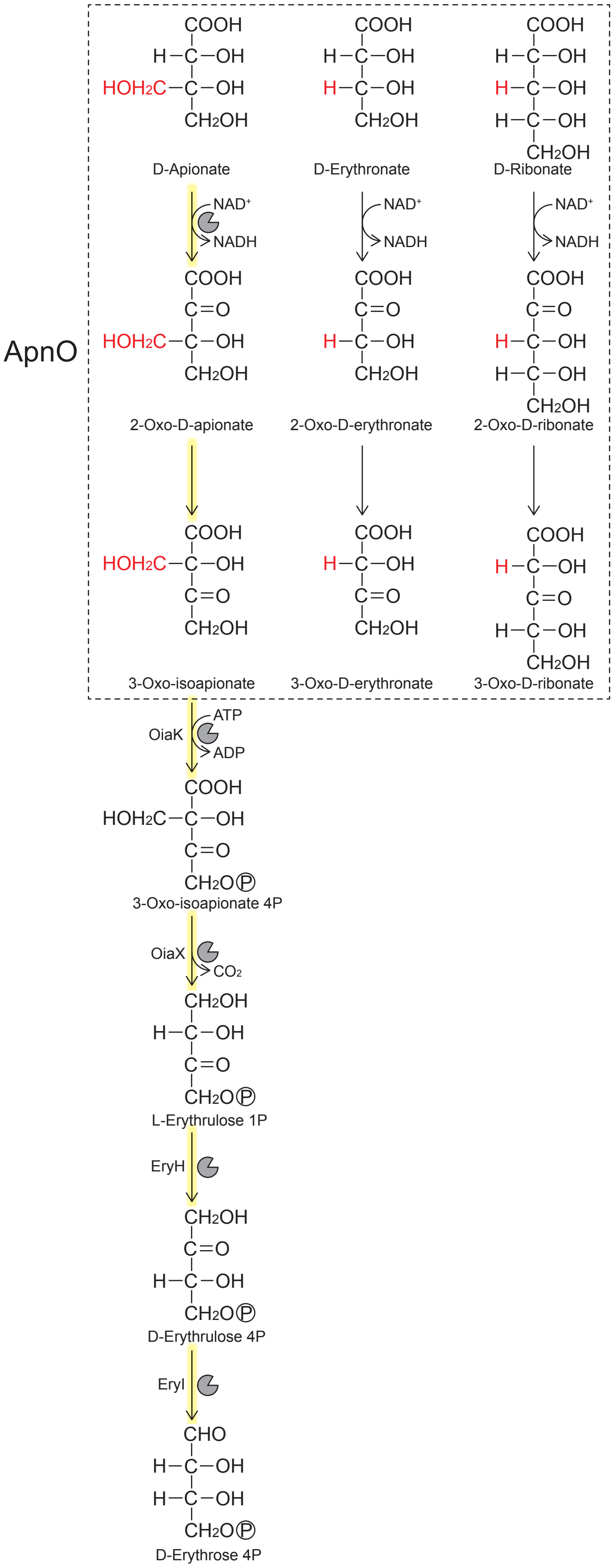

Figure S3. Schematic conversion of D-apionate to D-erythrose 4P in oxidative route 1 of D-apionate metabolism. Dashed-line box indicates potential reactions of D-erythronate and D-ribonate by ApnO. Migration of chemical group is colored in red.

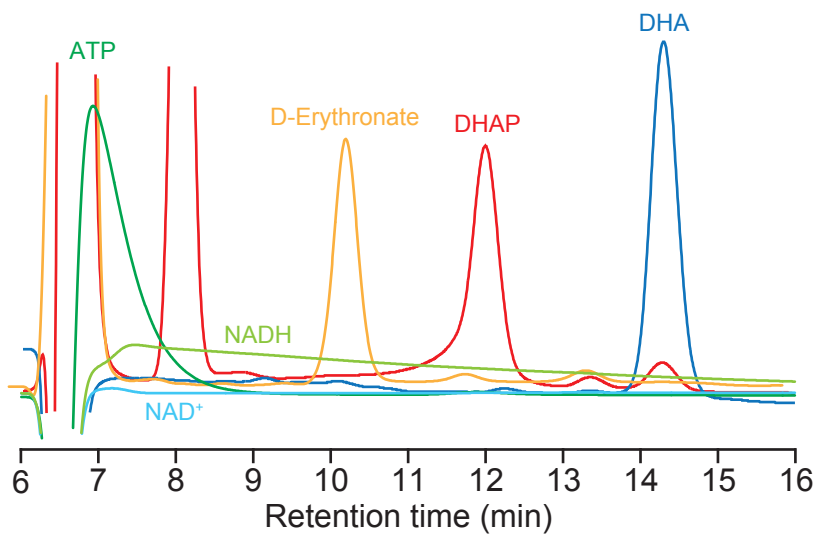

Figure S4. Elution profile of each component in reaction mixture of D-erythronate to DHAP (or DHA).

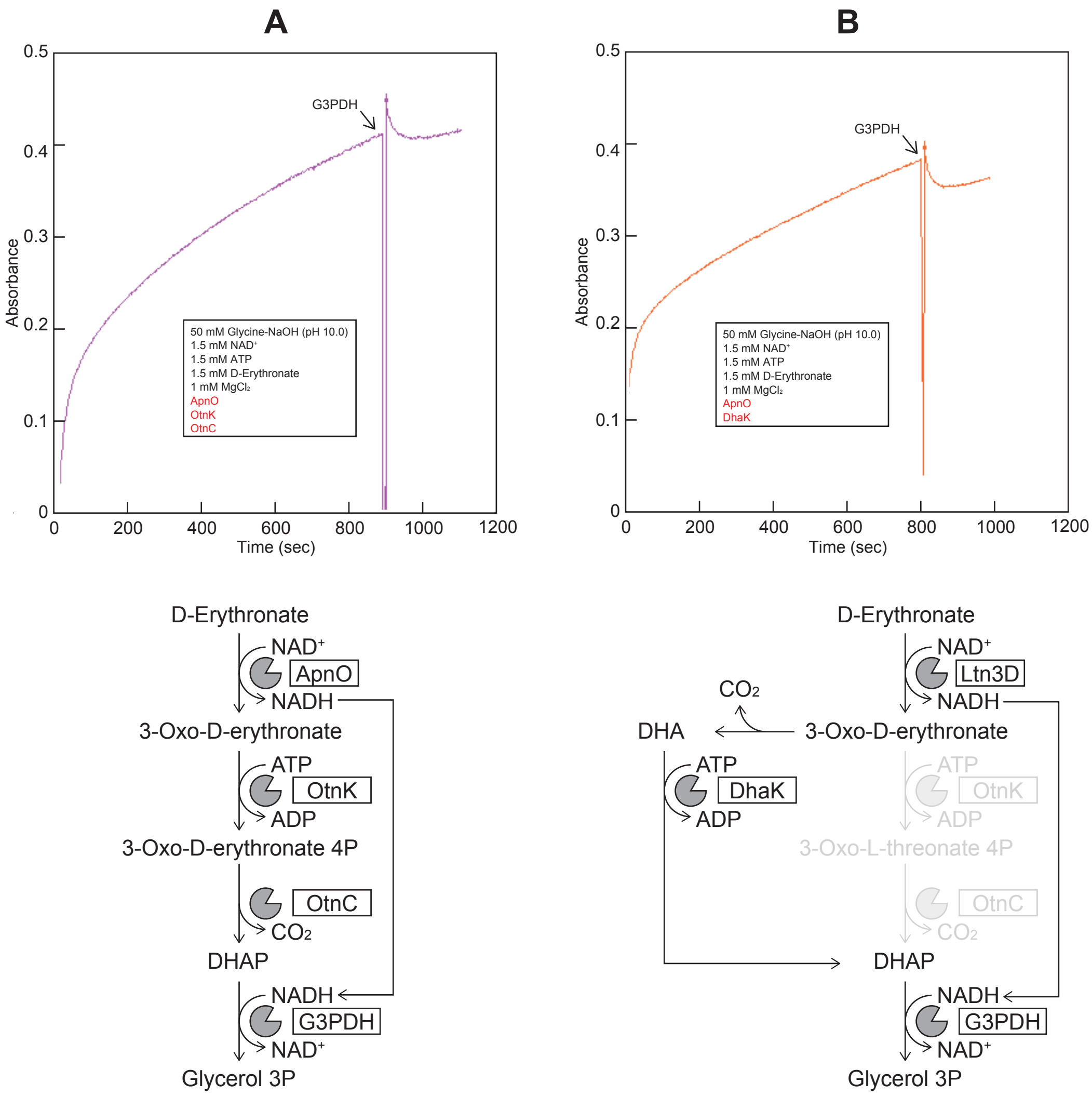

Figure S5. Coupled-enzyme spectrophotometric assay for AaApnO.
The initial reaction mixture is shown in box.

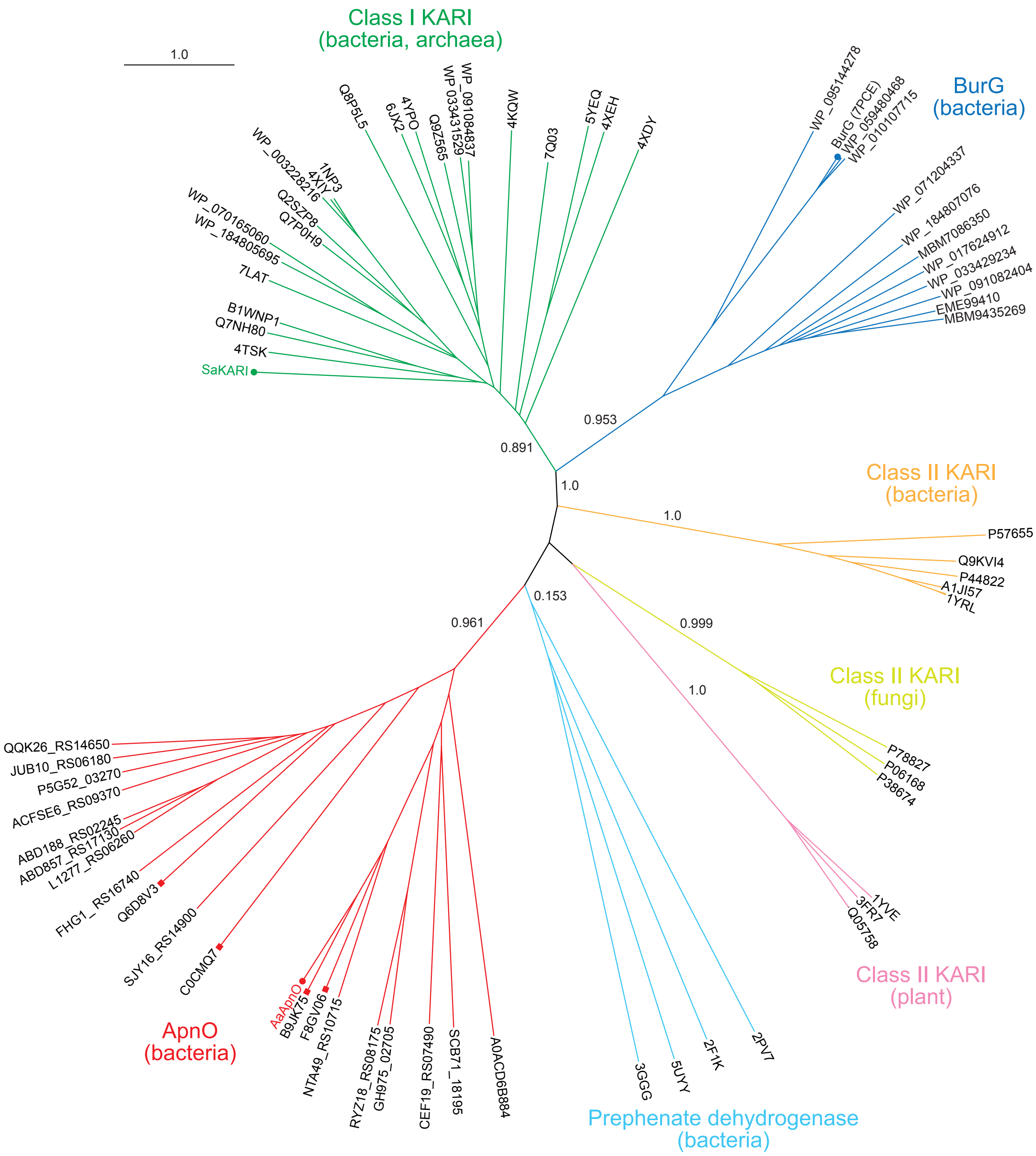

Figure S6. Extended phylogenetic tree of ApnO and its structural homologs.
The numbers at the nodes are bootstrapping values according to neighbor joining (1,000 replicates) for reliability of the different groups. The scale bar shows amino acid substitutions per site. Circles at the end of each branch are used for discussions in the text. Squares at the end each branch in ApnO subclass indicate the enzymes that have been functionally characterized by Gerlt and coworkers (5).

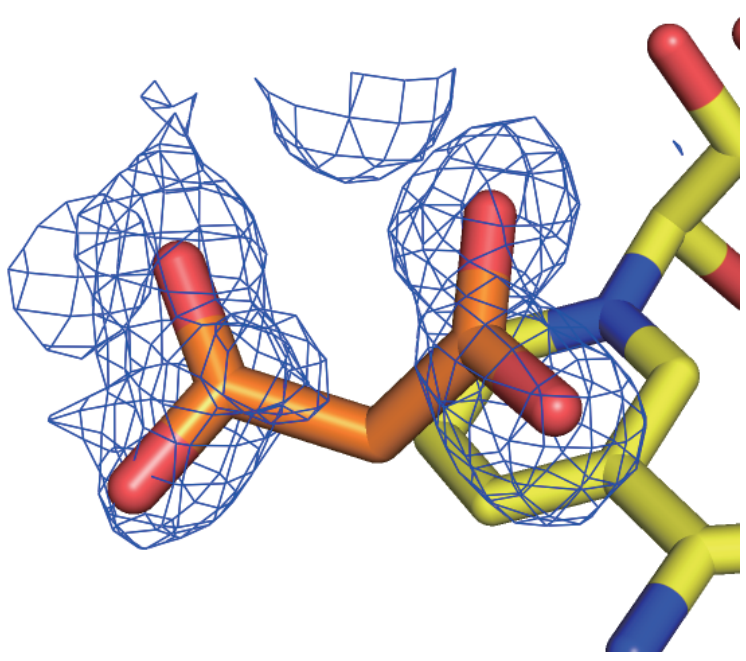

Figure S7. Electron density of the F_o-F_c omit map, contoured at 1.0 σ, and models of malonate. Only the electron densities corresponding to two the carboxyl groups of malonate are visualized.

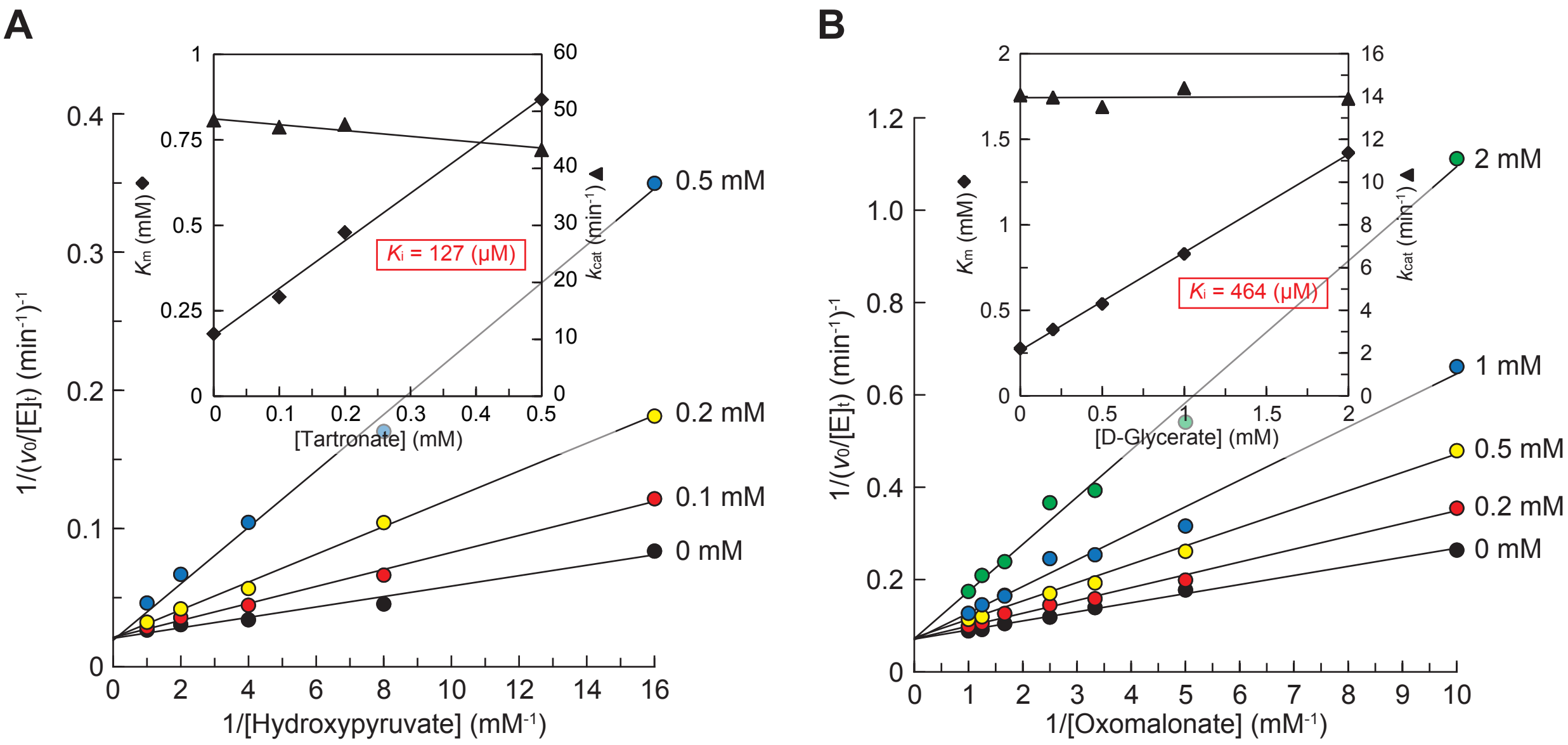

Figure S8. Lineweaver-Burk competitive inhibition by tartronate (A) and D-glycerate (B) in the reductive direction.

Hydroxypyruvate and oxomalonate concentrations were changed with fixed concentrations of inhibitor, which are indicated. The inset is K_m and k_{cat} values versus [inhibitor].

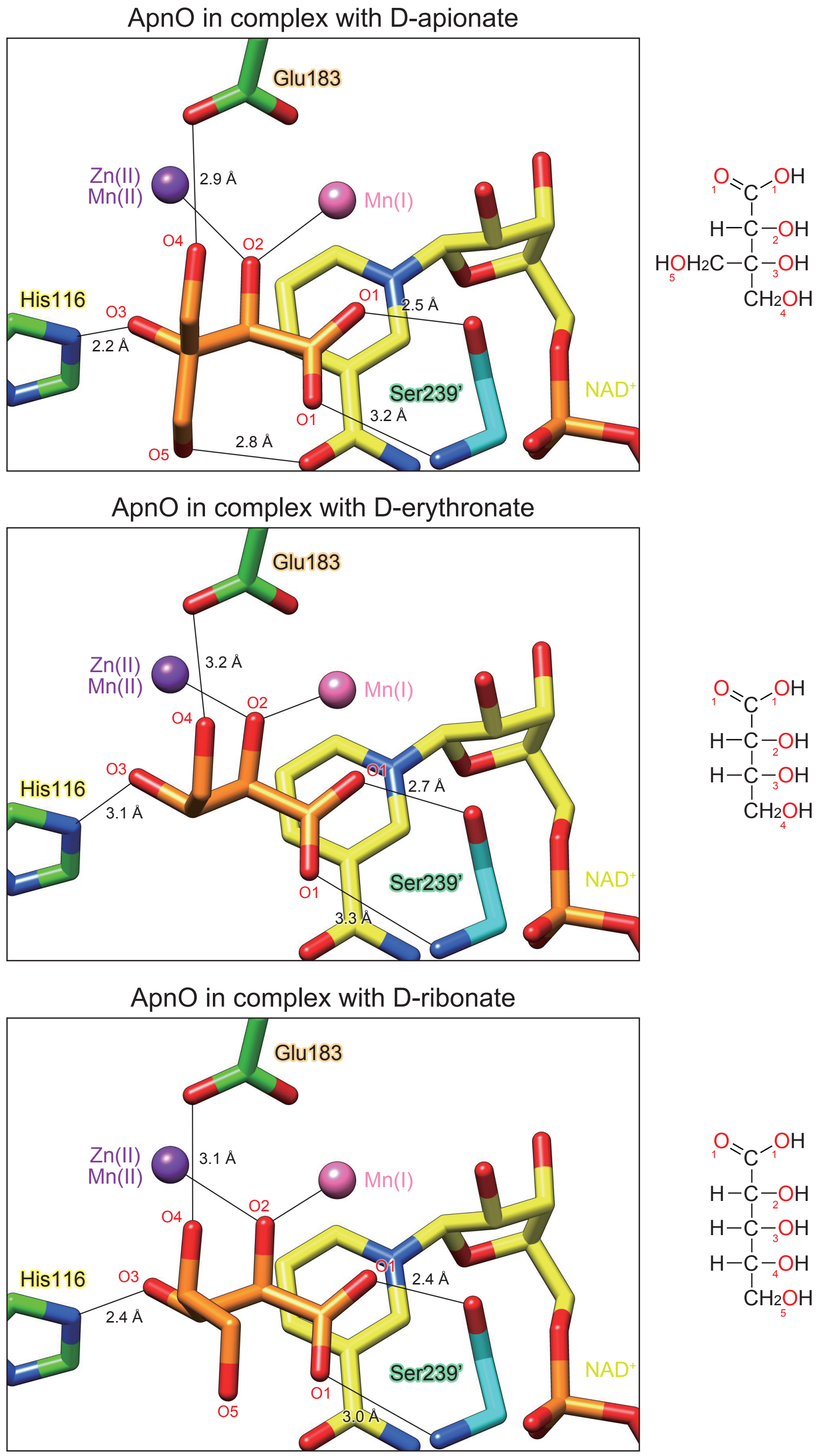

Figure S9. Docking model of AaApnO with substrate.
Intereaction between O5 atom and N7* atom of NAD^+ is found only in D-apionate.

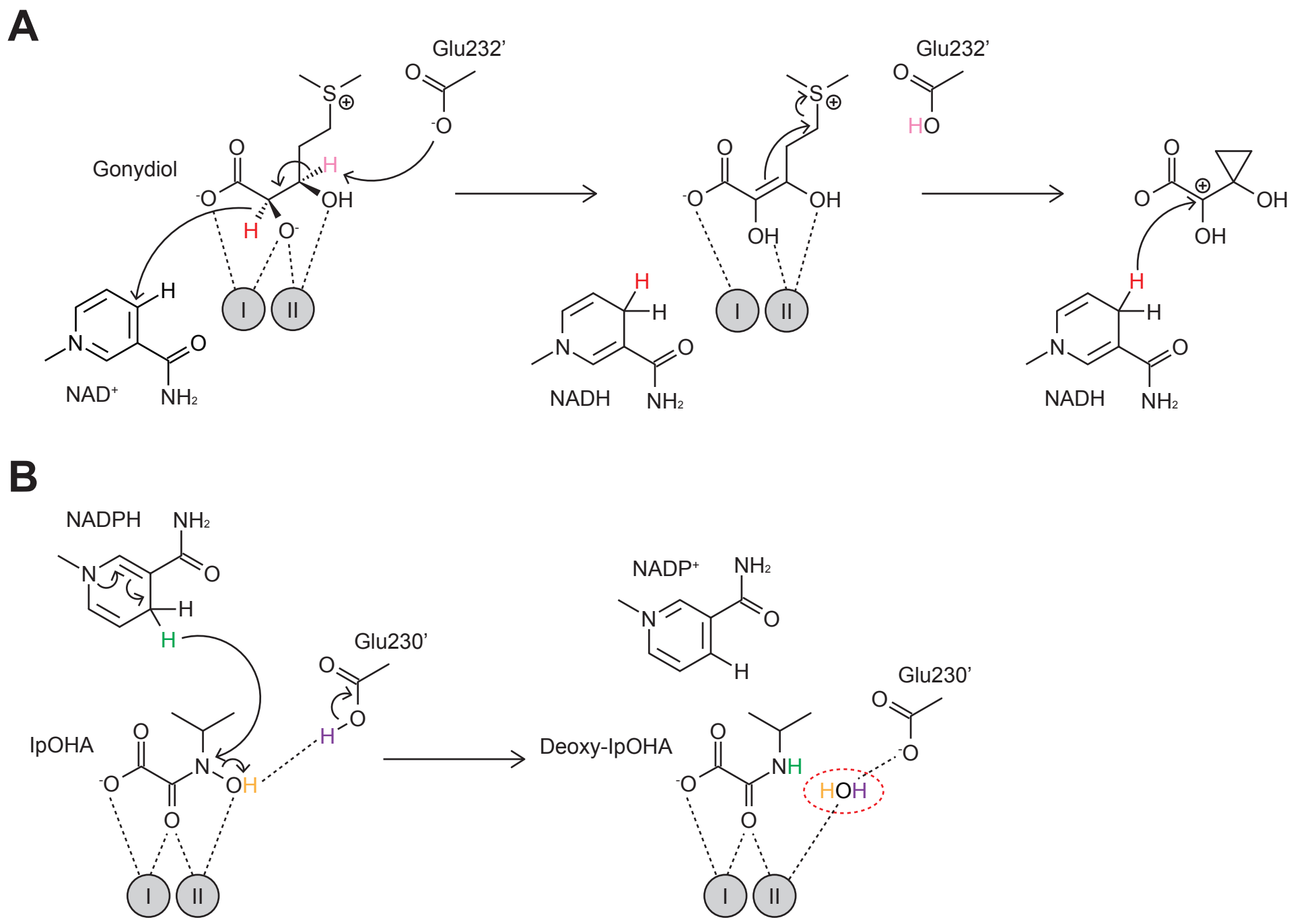

Figure S10. Comparison of reaction mechanism between ApnO, KARI, and BurG.
(A) Proposed reaction mechanism of BurG (ref. 15). (B) Proposed reaction mechanism of KARI for the reduction of IpOHA by NADPH (ref. 24). A red-colored water moleclue correspond to Fig. 7C.

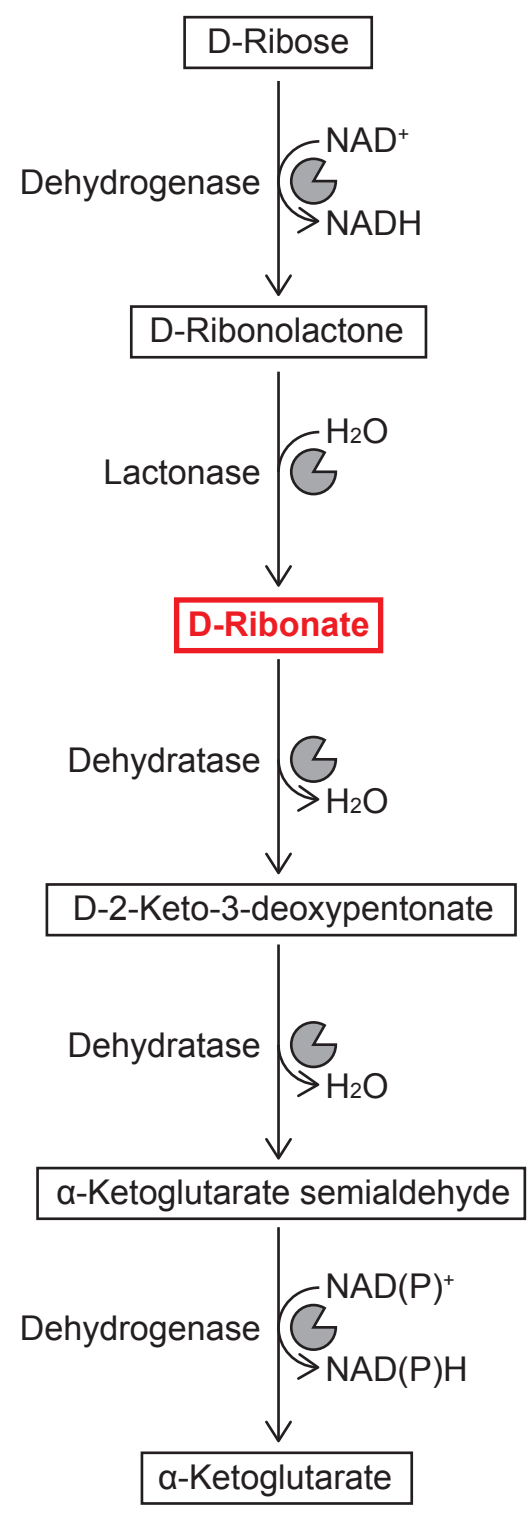

Figure S11. Metabolic pathway of D-ribose in archaea.
This is partially homologous to non-phosphorylating pathway to the unique glycolytic pathway from archaea.

Table S1. Lists of crystal structures of KARI and BurG.

PDB ID	Organism source	Domain	Site I	Site II	Ligand(s)		Class	References
					Coenzyme	Inhibitor		
1YVE	Spinach		Mg^{2+}	Mg^{2+}	NADPH	IpOHA		*EMBO J* 1997, 16, 3405-3415
1QMG			Mn^{2+}	Mn^{2+}	ADP-ribose	DHMV		*Acta Cryst* 2000, D56, 389-397
3FR7		Plant	Mg^{2+}	Mg^{2+}				
3FR8	Japanese rice<br>(subunit A)<br>(subunit B)			Mg^{2+}	NADPH	(PEG)		*J Mol Biol* 2009, 389, 167-182
			Mg^{2+}	Mg^{2+}			II	
9BAG			Mg^{2+}	Mg^{2+}		JK-5-114		*Chemistry* 2025, 31, e202501158
1YRL	*Escherichia coli*							*Protein Sci* 2005, 14, 3089-3100
3ULK			Mg^{2+}	Mg^{2+}	NADP			*J Mol Biol* 2012, 424, 168-179
1NP3	*Pseudomonas aeruginosa*	Bacteria						*J Mol Biol* 2003, 328, 505-515
4KQW	*Slackia exigua*		Mg^{2+}	Mg^{2+}	NADP	(Tartrate)		*PNAS* 2013, 110, 10946-10951
4KQX			Mg^{2+}	Mg^{2+}	NAD	IpOHA		
4TSK	*Alicyclobacillus acidocaldarius*		Mg^{2+}	Mg^{2+}	NADP	(Tartrate)		*Metab Eng* 2014, 26, 17-22
4XDY	Uncultured archaeon	Archaea	Mg^{2+}	Mg^{2+}	NADH	IpOHA		
4XEH	*Ignisphaera aggregans*							*Biochem J* 2015, 468, 475-484
4XDZ		Bacteria	Mg^{2+}	Mg^{2+}	NADPH	40E		
4XIY	*Azotobacter vinelandii*		Fe^{2+}	Mg^{2+}				
4YPO	*Mycobacterium tuberculosis*		Mg^{2+}	Mg^{2+}				*FEBS J.* 2016, 283, 1184-1196
5YEQ	*Sulfolobus acidocaldarius*			Mg^{2+}				*Sci Rep* 2018, 8, 7176
7Q03	*Methanothermococcus thermolithotrophicus*	Archaea	Mg^{2+}	Mg^{2+}	NADP			*Biomolecules* 2021, 11, 1679
7Q07					NADP	(Tartrate)		
7TOC	*Candida auris*	Fungi	Mg^{2+}	Mg^{2+}	NADPH	(Malonate)	I	Structural genomics
7LAT			Mg^{2+}	Mg^{2+}				
8SWM			Mg^{2+}	Mg^{2+}		AL		
8SXD	*Camphylobacter jejuni*		Mg^{2+}	Mg^{2+}	NADP	HMKB		*ACS Catal* 2024, 14, 6827-6841
8UPQ			Mg^{2+}	Mg^{2+}		DHIV		
8UPP			Mg^{2+}	Mg^{2+}	NADPH	Hoe704		
9B9P			Mg^{2+}	Mg^{2+}		JK-5-114		*Chemistry* 2025, 31, e202501158
9BAE	*Staphylococcus aureus*	Bacteria	Mg^{2+}	Mg^{2+}		JK-5-115		
5W3K			Mg^{2+}	Mg^{2+}	NADPH	CPD		*Chemistry* 2017, 23, 18289-18295
6AQJ			Mg^{2+}	Mg^{2+}	NADPH	IpOHA		
6L2I	*Streptococcus pneumoniae*		Mg^{2+}	Mg^{2+}	NADP			*Crystals* 2019, 9, 551
6JX2	*Corynebacterium glutamicum*		Mg^{2+}	Mg^{2+}				*J Agric Food Chem* 2019, 67, 8527-8535
7PCL (BurG)	*Burkholderia thailandensis*		Mg^{2+}	Mg^{2+}	NAD^+	CPD		*Nat Chem* 2022, 14, 884-890

Table S2. Primers used in the present study

Primer	Sequence
Cloning of AaApnO gene into pQE-80L[1]	
P1 (BamHI)	5′-catggatccACTTCGATCGCCCTGTTCGGCGCCGGTGGC-3′
P2 (HindIII)	5′-attaagcttAGGTGATGCGGCGGATGGAGGCGGCCAGC-3′
Site-directed mutagenesis in AaApnO gene[2]	
P3 (D68A-F)	5'-GTACCGGcCACGCTGATCGGCAAGGTTGCC-3'
P4 (D68A-R)	5'-CAGCGTGgCCGGTACGGCCAGCACCACCAC-3'
P5 (D92A-F)	5'-GTGCTGgcCGCGGCGGCCCCGTTTGCCGGC-3'
P6 (D92A-R)	5'-CGCCGCGgcCAGCACGACGACGATGGTGCC-3'
P7 (H113A-F)	5'-CGTCACCgccCCGTGCCATCCGCCGATCTTC-3'
P8 (H113A-R)	5'-GCACGGggcGGTGACGAAGTAGGTCAGGTC-3'
P9 (H116A-F)	5'-CCGTGCgccCCGCCGATCTTCAACGACG-3'
P10 (H116A-R)	5'-CGGCGGggcGCACGGATGGGTGACGAAGTAGGTC-3'
P11 (F133A-F)	5'-GACTTCgcCGGTGGCATCGCCGCCAAGCAG-3'
P12 (F133A-R)	5'-GCCACCGgcGAAGTCGCGCTTGGCGTCCATC-3'
P13 (E183A-F)	5'-CTTCTGGcGCCCGGCCTGTCCGAAACCGTC-3'
P14 (E183A-R)	5'-GCCGGGCgCCAGAAGGGCCATCTGCTCCAC-3'
P15 (S187A-F)	5'-GGCCTGgCCGAAACCGTCTGCGCCAGCCTG-3'
P16 (S187A-R)	5'-GGTTTCGGcCAGGCCGGGCTCCAGAAGGGC-3'
P17 (E188A-F)	5'-CTGTCCGcAACCGTCTGCGCCAGCCTGCTG-3'
P18 (E188A-R)	5'-GACGGTTgCGGACAGGCCGGGCTCCAGAAG-3'
P19 (F218A-F)	5'-CGCGACgagCTTCTCGGCCACATGAACGTG-3'
P20 (F218A-R)	5'-GAGAAGctcGTCGCGTGCGGCTTCCTTCGG-3'
P21 (H222A-F)	5'-CTCGGCgcCATGAACGTGCTCGGCTCGGTG-3'
P22 (H222A-R)	5'-GTTCATGgcGCCGAGAAGGAAGTCGCGTGC-3'
P23 (V225A-F)	5'-ATGAACGcGCTCGGCTCGGTGATCTTCGAG-3'
P24 (V225A-R)	5'-GCCGAGCgCGTTCATGTGGCCGAGAAGGAAG-3'
P25 (L226A-F)	5'-AACGTGgcCGGCTCGGTGATCTTCGAGGAG-3'
P26 (L226A-R)	5'-CGAGCCGgcCACGTTCATGTGGCCGAGAAG-3'
P27 (F238A-F)	5'-GGCGTGgcCTCCGACGCCTGCAACAAGGCG-3'
P28 (F238A-R)	5'-GTCGGAGgcCACGCCGGGTGTCTCCTCGAAG-3'
P29 (S239A-F)	5'-GTGTTCgCCGACGCCTGCAACAAGGCGATC-3'
P30 (S239A-R)	5'-GGCGTCGGcGAACACGCCGGGTGTCTCCTC-3'
P31 (C242A-F)	5'-GACGCCgcCAACAAGGCGATCGAGTTCGGC-3'
P32 (C242A-R)	5'-CTTGTTGgcGGCGTCGGAGAACACGCCGGG-3'
[1] Lower case letters indicate additional bases for introducing the underlined digestion sites of restriction enzymes in parentheses.	
[2] F and R indicate forward and reverse primers, respectively. Underlined areas indicate mutated regions (codons).	
